# Enhanced Recovery of Antioxidant Compounds from Hazelnut (*Corylus avellana* L.) Involucre Based on Extraction Optimization: Phytochemical Profile and Biological Activities

**DOI:** 10.3390/antiox8100460

**Published:** 2019-10-08

**Authors:** Marius Emil Rusu, Ionel Fizeșan, Anca Pop, Ana-Maria Gheldiu, Andrei Mocan, Gianina Crișan, Laurian Vlase, Felicia Loghin, Daniela-Saveta Popa, Ioan Tomuta

**Affiliations:** 1 Department of Pharmaceutical Technology and Biopharmaceutics, Faculty of Pharmacy, “Iuliu Hatieganu” University of Medicine and Pharmacy, 8 Victor Babes, 400012 Cluj-Napoca, Romania; marius.e.rusu@gmail.com (M.E.R.); laurian.vlase@umfcluj.ro (L.V.); tomutaioan@umfcluj.ro (I.T.); 2 Department of Toxicology, Faculty of Pharmacy, “Iuliu Hatieganu” University of Medicine and Pharmacy, 8 Victor Babes, 400012 Cluj-Napoca, Romania; ionel.fizesan@umfcluj.ro (I.F.); floghin@umfcluj.ro (F.L.); dpopa@umfcluj.ro (D.-S.P.); 3 Department of Pharmaceutical Botany, Faculty of Pharmacy, “Iuliu Hatieganu” University of Medicine and Pharmacy, 8 Victor Babes, 400012 Cluj-Napoca, Romania; mocan.andrei@umfcluj.ro (A.M.); gcrisan@umfcluj.ro (G.C.)

**Keywords:** hazelnut involucre, antioxidants, polyphenols, phytosterols, biological activity, experimental design, LC-MS, turbo-extraction, Ultra-Turrax, enzymatic inhibition

## Abstract

Tree nut by-products could contain a wide range of phytochemicals, natural antioxidants, which might be used as a natural source for dietary supplements. The aim of the present study was to evaluate the phenolic and sterolic composition, as well as the antioxidant and other biological activities, of hazelnut involucre (HI) extracts. Experimental designs were developed in order to select the optimum extraction conditions (solvent, temperature, time) using turbo-extraction by Ultra-Turrax for obtaining extracts rich in bioactive compounds. Qualitative and quantitative analyses were performed by LC-MS and LC-MS/MS and they revealed important amounts of individual polyphenols and phytosterols, molecules with antioxidant potential. The richest polyphenolic HI extract with the highest antioxidant activity by TEAC assay was further evaluated by other in vitro antioxidant tests (DPPH, FRAP) and enzyme inhibitory assays. Additionally, the cytotoxic and antioxidant effects of this extract on two cancerous cell lines and on normal cells were tested. This is the first study to analyze the composition of both hydrophilic and lipophilic bioactive compounds in HI extracts. Our findings reveal that this plant by-product presents strong biological activities, justifying further research, and it could be considered an inexpensive source of natural antioxidants for food, pharmaceutical, or cosmetic industry.

## 1. Introduction 

The interest in traditional bioactive herbal compounds with less detrimental effects on human body than their synthetic counterparts is growing. Many clinical trials and cohort studies showed that consumption of foods (vegetables, fruits, nuts) rich in biologically active molecules with demonstrated antioxidant capacity, through different mechanisms of actions, has the potential to protect against cardiometabolic diseases, neurodegenerative disorders, cancer, or age-related sarcopenia and frailty [1,2,3,4,5]. Increased intake of antioxidants from tree nuts and peanuts influences risk factors associated with aging and can extend health span and lifespan [6].

Hazelnut (*Corylus avellana* L.) is known for the nutritional and therapeutic properties of its fruits, rich sources of healthy fatty acids, vitamins, minerals, and polyphenols [7,8]. Polyphenols, common secondary plant metabolites characterized by several hydroxyl groups linked to a phenol ring, can act synergistically with other phytochemicals to lower the oxidation and inflammation processes which may trigger many pathological conditions or age-related chronic diseases [9,10,11]. These biologically active molecules donate electrons or hydrogen atoms to reactive free radicals preventing lipid oxidation or cellular damage and acting as natural antioxidants with many health benefits [12]. In addition, plant polyphenols act as signaling molecules and can participate in many enzymatic pathways [13].

Recently, special interest was given to tree nut by-products, waste plant matrices, with great potential as sources of biologically active compounds. As little is known about the biochemical profile of hazelnut involucre (HI) [14], we intended to extend the knowledge about this promising by-product. In a previous study, we successfully applied a d-optimal experimental design to optimize the bioactive compound extraction from walnut septum, another tree nut by-product [15]. In that experiment, acetone was more efficient than ethanol to extract both polyphenols and phytosterols from the plant matrix. The objectives of this study were the optimization of the extraction process in order to identify the optimal extraction conditions for attaining rich bioactive compound extracts from HI and the emphasizing of key biological activities for the optimally selected extract, which would justify further research on this plant matrix. The first aim of our study was the analysis of phenolic and phytosterol compounds from HI based on experimental designs. Turbo-extraction (TE) by Ultra-Turrax, an efficient and economical extraction method, with higher outcomes compared to other methods, was chosen [15]. Three extraction variables, stirring time, pH, and percentage of water in acetone, were selected to define the optimal extraction conditions. LC-MS analysis was chosen for the identification and quantification of several individual polyphenols and phytosterols from involucre. Based on the obtained results, optimal conditions were selected to achieve the HI extract with the highest content of polyphenolic compounds and the highest antioxidant activity (AA) determined by Trolox equivalent antioxidant capacity (TEAC) assay. The biological activities of this optimal extract were further examined by other in vitro antioxidant assays (DPPH and FRAP), for enzymatic inhibitory capacity (tyrosinase and α-glucosidase), and a potential cytotoxic effect on lung and breast cancer cell lines. 

## 2. Materials and Methods 

### 2.1. Chemicals 

2,2-Diphenyl-1-2,4,6-trinitro-phenyl hydrazine (DPPH), 2,4,6-tris(2-pyridyl)-S-triazine (TPTZ) (≥99%), 3,4-dihydroxy-l-phenylalanine (l-DOPA) (≥98%), 6-hydroxy-2,5,7,8-tetramethylchromane-2-carboxylic acid (Trolox) (≥97%), 2,2′-azino-bis(3-ethylbenzothiazoline-6-sulfonic acid) diammonium salt (ABTS) (≥98%), dimethyl sulfoxide (DMSO) (≥99%), ferric chloride, hydrogen peroxide 30% solution, kojic acid, mushroom tyrosinase, *N*-acetyl-l-cysteine (≥99%), phosphate buffer, *p*-nitrophenyl-β-d-glucuronide (PNPG), sodium carbonate, vanillin (99%), resazurin, neutral red, 2,7 dichloro-fluorescein diacetate (DCFH-DA), acetic acid (≥99%) were acquired from Sigma Aldrich (Schnelldorf, Germany). Acetone, Folin–Ciocâlteu reagent, hydrochloric acid (HCl) (37%), sodium phosphate (≥99%) were acquired from Merck (Darmstadt, Germany). α-Glucosidase solution (*Saccharomyces cerevisiae*, EC 3.2.1.20) was obtained from Sigma (Darmstadt, Germany). Aluminum chloride (≥98%) was acquired from Carl Roth (Karlsruhe, Germany), Dulbecco’s modified Eagle medium (DMEM) from Gibco (Paisley, UK), and fetal bovine serum (FBS) from Sigma Aldrich (Steinheim, Germany).

All solvents were of LC grade and all reagents were of analytical grade. 

The standards used for both spectrophotometric and liquid chromatography–mass spectrometry (LC-MS) analyses were: apigenin (≥95%), caffeic acid (≥98%), brassicasterol (≥98%), caftaric acid (≥97%), (+)-catechin (≥96%), chlorogenic acid (≥95%), (−)-epicatechin (≥90%), fisetin (≥98%), gentisic acid (≥98%), l-glutathione (≥98%), hyperoside (quercetin 3-d-galactoside) (≥97%), isoquercitrin (quercetin 3-β-d-glucoside) (≥98%), kaempferol (≥97%), luteolin (≥98%), myricetin (≥96%), patuletin (≥98%), *p*-coumaric acid (≥98%), protocatechuic acid (3,4-dihydroxybenzoic acid) (≥97%), quercetin (≥95%), quercitrin (quercetin 3-rhamonoside) (≥78%), rutoside (quercetin-3-*O*-rutinoside) (≥97%), syringic acid (≥95%), vanillic acid (≥97%), campesterol (~65%), ergosterol (≥95%), stigmasterol (~95%) acquired from Sigma-Aldrich, ferulic acid (≥99%), gallic acid (≥98%) acquired from Merck (Darmstadt, Germany), beta-sitosterol (≥80%), sinapic acid (≥98%) acquired from Carl Roth (Karlsruhe, Germany).

### 2.2. Plant Samples 

Hazelnut (*Coryllus avelana* L.) involucre of high quality was provided by an organic orchard in Cluj County, Romania. In the autumn of 2017, hazelnuts were harvested and separated from the involucre. The by-product was dried in the shade for three days at 18–22 °C, and then it was delivered to the Faculty of Pharmacy, “Iuliu Hatieganu” University of Medicine and Pharmacy Cluj-Napoca, Romania. 

#### Preparation of the Extracts 

The involucre was ground in a coffee grinder (Argis, RC-21, Electroarges SA, Curtea de Arges, Romania) for 5 min and the powder was screened through a 200 µm Retsch sieve. HI was weighed (2 g) and mixed with the extraction solvent (20 mL) in Falcon tubes. TE was accomplished using an Ultra-Turrax homogenizer (T 18; IKA Labortechnik, Staufen, Germany) for 1 to 3 min (12,000 rpm) and a Vortex RX-3 (Velp Scientifica, Usmate, Italy) for 2 min. The homogenates were centrifuged (Hettich, Micro 22R, Andreas Hettich GmbH & Co., Tuttlingen, Germany) 15 min at 5000 rpm. The supernatant was carefully separated and, using a rotary evaporator (Hei-VAP, Heidolph Instruments GmbH & Co., Schwabach, Germany), the solvent was removed under vacuum at 45 °C. The dry residue was taken up in water, placed in amber glass vials, and lyophilized (Advantage 2.0, SP Scientific, Warminster, PA, USA). After lyophilization, the samples (weight between 6 and 520 mg) were stored at room temperature. For further analyses, the lyophilized extracts were dissolved in 70% EtOH (10 mg/mL), if not specified otherwise. All assays were performed in triplicate. 

Two d-optimal experimental designs, implemented by Modde software, version 11.0 (Sartorius Stedim Data Analytics AB, Umeå, Sweden), were developed for the extraction process optimization. Three factors, stirring time, pH, and percentage of water in acetone, were the independent variables for both d-optimal experimental designs. The total phenolic content (TPC), total flavonoid content (TFC), condensed tannin content (CTC), and the AA-TEAC were the dependent variables in the first experimental design used in the screening step (Table 1). The extracts were prepared according to this first experimental design. The individual concentrations of the bioactive compounds quantified by LC/MS methods were the dependent variables in the second experimental design used in the optimization step (Table 2).

### 2.3. Determination of Total Bioactive Compounds 

#### 2.3.1. Total Phenolic Content 

The TPC of the HI extracts was assessed by Folin–Ciocâlteu (FC) spectrophotometric method as previously reported [15,16]. A Synergy HT multi-detection microplate reader with 96-well plates (BioTek Instruments, Inc., Winooski, VT, USA) was used for high sample throughput. At first, 20 µL of each sample (HI extracts diluted five times) were mixed with 100 µL of FC reagent (diluted 1:10) and, after 3 min, 80 µL of sodium carbonate solution (7.5% *w/v*) was added. The mixtures were incubated in the dark at room temperature for 30 min. The absorbance was measured at 760 nm against a solvent blank. Gallic acid was used as a reference standard, and the TPC was expressed as gallic acid equivalents (GAE) per dry weight (dw) of involucre (mg GAE/g dw).

#### 2.3.2. Total Flavonoid Content 

The TFC of the HI extracts was assessed according to a method previously described [17]. In a 96-well plate, 100 µL of sample extract was added to 100 µL of 2% AlCl_3_ aqueous solution. The plate was incubated in the dark at room temperature for 15 min and the absorbance was measured at 420 nm against a solvent blank. The TFC was expressed as quercetin equivalents (QE) per dw of involucre (mg QE/g dw).

#### 2.3.3. Condensed Tannin Content 

The CTC in HI extracts was assessed according to a modified version of the vanillin assay formerly described [18]. In a 96-well plate, 50 µL of HI sample was added to 250 µL 0.5% vanillin in 4% concentrated HCl in methanol. After the plate was incubated in the dark at 30 °C for 20 min, the absorbance was measured at 500 nm against a solvent blank. The CTC was expressed as catechin equivalents (CE) per dw of involucre (mg CE/g dw).

### 2.4. Determination of the Antioxidant Activity

#### 2.4.1. TEAC Assay 

The antiradical activity of HI extracts was assessed according to the TEAC assay previously described [17]. Briefly, 20 µL of the sample was mixed with 200 µL of ABTS radical solution, incubated for 6 min, and the absorbance of the mixture was measured at 760 nm. The scavenging activity against ABTS radical cation was calculated and used to plot the Trolox calibration curve. The AA according to this assay was expressed as Trolox equivalents (TE) per dw involucre (mg TE/g dw). This assay was used during the screening phase of the study for the evaluation of AA of all HI extracts obtained within the experimental plan in order to optimize the extraction method. 

#### 2.4.2. DPPH Radical Scavenging Activity 

The antiradical activity of HI extracts against the free radical DPPH was measured using a method previously described [19]. In a 96-well plate, 30 µL of sample solution was mixed with a 0.004% methanol solution of DPPH, and then incubated in the dark for 30 min. The absorbance was measured at 517 nm against a solvent blank. Trolox was used as a reference standard and the results were expressed as TE per dw extract (mg TE/g dw extract). This assay was performed only on the richest polyphenolic HI extract.

#### 2.4.3. FRAP Assay 

The reduction capacity of the HI extract was assessed by ferric reducing antioxidant power (FRAP) assay (analyzes the reduction of Fe^3+^-TPTZ to the blue-colored Fe^2+^-TPTZ) using a slight modified previously described method [20]. Briefly, a quantity of 25 µL sample was incubated with 175 µL FRAP reagent (300 mM acetate buffer, pH 3.6: 10 mM TPTZ in 40 mM HCl: 20 mM FeCl_3_·6H_2_O in 40 mM HCl, 10:1:1, *v/v/v*) in the dark for 30 min. The absorbance was measured at 593 nm and the results were expressed as TE per dw extract (mg TE/g dw extract). This assay was done only on the richest polyphenolic HI extract.

### 2.5. Phytochemical Analysis by LC-MS 

The phytochemical profiles of the lyophilized HI extracts were analyzed by LC-MS. For this assay, an Agilent 1100 HPLC Series system (Agilent, Santa Clara, CA, USA), equipped with degasser, binary gradient pump, column thermostat, autosampler, and UV detector, coupled with an Agilent Ion Trap 1100 SL mass spectrometer (LC/MSD Ion Trap VL) were used.

#### 2.5.1. Identification and Quantification of Individual Polyphenolic Compounds 

An LC-MS method previously described [21,22] was used for the identification of individual polyphenols in the HI extracts. The 18 external standards were: apigenin, caffeic acid, caftaric acid, chlorogenic acid, ferulic acid, fisetin, gentisic acid, hyperoside, isoquercitrin, kaempferol, luteolin, myricetin, patuletin, *p*-coumaric acid, quercetin, quercitrin, rutoside, and sinapic acid (Table S1). In brief, the chromatographic separation was performed on a reverse-phase analytical column (Zorbax SB-C18, 100 mm × 3.0 mm i.d., 3.5 µm particles) with a mixture of methanol/acetic acid 0.1% (*v/v*) as mobile phase and a binary gradient. The elution started with a linear gradient, beginning with 5% methanol and ending at 42% methanol at 35 min, isocratic elution followed with 42% methanol for the next 3 min, rebalancing with 5% methanol in the next 7 min. The flow rate was 1 mL/min, the injection volume was 5 µL, and the column temperature was 48 °C. The detection procedure was performed on both UV and MS mode. The UV detector was set at 330 nm until 17 min (for the detection of polyphenolic acids), then at 370 nm until the end of analysis time (for the detection of flavonoids and their aglycones). The MS system operated using an electrospray ion (ESI) source in negative mode (capillary 3000 V, nebulizer 60 psi (nitrogen), dry gas temperature 360 °C, and dry nitrogen gas at 12 L/min). 

Another LC-MS method (LC-MS method II) previously described [23] was used to detect the other six polyphenols (epicatechin, catechin, syringic acid, gallic acid, protocatechuic acid, and vanillic acid) in HI extracts (Table S2). The chromatographic separation was accomplished on the same analytical column and in the same chromatographic conditions as mentioned before but with a slightly different binary gradient (start: 3% methanol; at 3 min: 8% methanol; at 8.5 min: 20% methanol; at 10 min: rebalance column with 3% methanol). The detection of the compounds was performed on MS mode. All identified polyphenols were measured both in the HI non-hydrolyzed and hydrolyzed extracts (equal amounts of extract and 4 M HCl kept 30 min on 100 °C water bath) on the basis of their peak areas and comparison with a calibration curve of their corresponding standards. The results were expressed as micrograms of phenolic compounds per dw of involucre (μg/g dw).

#### 2.5.2. Identification and Quantification of Phytosterols 

The phytosterols in the HI extracts were determined according to an LC-UV-MS/MS method previously described [24]. In brief, apparatus and chromatographic analytical column were the same, but elution of compounds was performed in an isocratic mode, mobile phase containing acetonitrile/methanol (90:10, *v/v*), with a flow rate of 1 mL/min at 45 °C and 5 μL injection volume. For the detection of the analytes, the same ion trap mass spectrometer was used, fitted with an atmospheric pressure chemical ionization (APCI) interface in a positive mode. Operating conditions (dry nitrogen gas temperature 325 °C at a flow rate of 7 L/min, nebulizer pressure 60 psi, capillary voltage −4000 V) were adjusted for achieving maximum sensitivity values. 

The full identification of compounds was performed by comparing the retention times and mass spectra with five external standards (ergosterol, brassicasterol, stigmasterol, campesterol, beta-sitosterol) (Table S3). Under the ionization conditions used for their determination, all sterols lose a water molecule and their pseudo-molecular ions are in the form M−H_2_O+H^+^ [25]. The multiple reactions monitoring (MRM) analysis mode was used for detection in order to avoid or reduce the interference from the background. The quantification was performed based on the intensity of major daughter ions in the mass spectra [25]. The results were expressed as micrograms of phytosterols per dw involucre (μg/g dw).

The Agilent ChemStation (vA09.03) and DataAnalysis (v5.3) software were used for the acquisition and investigation of chromatographic data. 

### 2.6. Selection and Biological Activities of the Optimal Hazelnut Involucre Extract

Based on the experimental data obtained during the screening and the optimization steps, the extract with the richest polyphenolic content and the best antioxidant activity by TEAC assay was further selected as the optimal HI extract in order to evaluate its biological effects. The antioxidant activities by DPPH and FRAP assays, the enzymatic inhibitory potential (tyrosinase and α-glucosidase), and the cytotoxicity and antioxidant effects on cellular cultures were further evaluated for this extract. All determinations were performed in triplicate on the lyophilized extract.

#### 2.6.1. Enzyme Inhibitory Activities

##### Tyrosinase Inhibitory Activity 

The tyrosinase inhibitory activity of HI extract was calculated using a slightly modified previously described method [26]. In a 96-well plate, four wells were designated (HI lyophilized extract dissolved in water containing 5% DMSO) as follows: (A) 46 U/mL (40 µL) mushroom tyrosinase (MT) in 66 mM phosphate buffer (PB), pH 6.6 (120 µL); (B) only PB (160 µL); (C) PB (80 µL), MT (40 µL) and the sample (40 µL); (D) PB (120 µL) and the sample (40 µL). After incubation at room temperature for 10 min, 2.5 mM L-DOPA prepared in PB (40 µL) was added in all wells. After room temperature incubation for 20 min, the absorbance was measured at 475 nm. The tyrosinase inhibitory activity was calculated using kojic acid as an external standard (0.01–0.10 mg/mL). The inhibition percentage of enzymatic activity was assessed by the following equation: [(A − B) − (C − D)] × 100/(A − B). The results were expressed as milligram kojic acid equivalents (KAE) per dw extract (mg KAE/g dw). 

##### α-Glucosidase Inhibitory Assay

The α-glucosidase inhibitory activity was performed using the protocol previously described [27]. Briefly, 50 µL sample solution (2 mg HI lyophilized extract/mL), 50 µL glutathione (0.5 mg/mL), 50 µL of 10 mM PNPG (*p*-nitrophenyl-β-d-glucuronide) solution, and 50 µL α-glucosidase solution in PB (pH 6.8) were mixed in a 96-wells microplate and incubated at 37 °C for 15 min. Similarly, the blank was prepared by adding a sample solution to all reaction reagents without the enzyme (α-glucosidase) solution. The reaction was finally stopped by adding 50 µL of 0.2 M sodium carbonate. The absorbance for sample and blank were read at 400 nm and then the absorbance of blank was subtracted from the absorbance of the sample.

#### 2.6.2. Biological Activities of HI Extract on Cell Lines 

##### Cell Culture

The normal human gingival fibroblasts (HGF) (CLS Cell Lines Service, Eppelheim, Germany), and the cancerous cell lines A549 (human lung adenocarcinoma) and T47D-KBluc (human breast cancer) (ATCC, Manassas, United States of America) were maintained in Dulbecco’s modified Eagle medium (DMEM) supplemented with 10% fetal bovine serum (FBS) on culture flasks at 37 °C in a humidified incubator with 5% CO_2_ supplementation. The medium was changed every two to three days and the cells were subcultured once they reached 70%–80% confluence.

##### Preparation of Extract Solutions

A 100 mg/mL stock solution was prepared in dimethyl sulfoxide (DMSO) from lyophilized extract. The stock solution was further diluted in DMSO to obtain working solutions of 0.25, 6.25, 12.5, 18.75, 25, 37.25, 50, and 75 mg/mL. These working solutions were then used to obtain the desired concentrations, ranging from 400 to 1 µg/mL, in the cell culture medium.

##### Viability Assays 

Cells were seeded in 96-well plates and left to attach for 24 h. Dead and unattached cells were washed with PBS while the remaining viable cells were further exposed for 24 h/48 h to the HI extract. Following the exposure, the cells were washed with PBS and viability was assessed by two complementary assays, Alamar Blue assay, and Neutral Red assay. 

Alamar Blue (AB) assay was used to measure the metabolic ability of exposed cells to convert resazurin, a non-fluorescent compound, to resorufin, a fluorescent product. The cells were exposed to a resazurin solution of 200 µM for 3 h and the fluorescence was measured at λ_excitation_ = 530/25, λ_emission_ = 590/35, using Synergy 2 multi-mode microplate reader. 

Neutral Red (NR) assay was used to measure the ability of the exposed cells to incorporate the supravital dye neutral red. The cellular uptake of this dye reveals the capacity of the cell to maintain pH gradients through the production of ATP. Briefly, 150 µL 40 μg/mL neutral red dye was added to each well. The cells were incubated with the dye at 37 °C for 2 h. Afterwards, the cells were washed twice with PBS and accumulated dye was extracted by adding a solution containing 50% ethanol, 49% water, and 1% glacial acetic acid to each well. Cell viability was determined by measuring the fluorescence at λ_excitation_ = 530/25, λ_emission_ = 620/40, using Synergy 2 multi-mode microplate reader. 

The experiments were performed with three biological replicates, each one including six technical replicates. The results were expressed as relative values compared to the negative control (100%) (cells exposed to culture medium containing 0.2% DMSO). 

IC_50_ values were calculated, when possible, from the dose-response curves obtained for each condition using a four-parameter logistic curve, in order to allow a comparison between the conditions tested.

##### Dichloro-Fluorescein Diacetate (DCFH-DA) Assay

The ability of the HI extract to protect against the oxidative stress in T47D-KBluc, A549, and HGF cells was monitored using the reactive oxygen species (ROS) sensitive 2,7 dichloro-fluorescein diacetate (DCFH-DA) dye. After a 24 h treatment with non-toxic HI extract concentrations, the cells were washed with PBS and further loaded with 50 µM DCFH-DA in Hanks’ balanced salt solution (HBSS) for 2 h. Following incubation, the excess of DCFH-DA was washed, and the cells were exposed to 250 µM H_2_O_2_ for 2 h. The conversion of DCFH-DA to the fluorescent compound dichlorofluorescein (DCF) was measured using Synergy 2 multi-mode microplate reader at λ_excitation_ = 485/20, λ_emission_ = 528/20. The potency of HI extracts, to mitigate the induction of oxidative stress after H_2_O_2_ treatment, was compared to N-Acetyl Cysteine (NAC) treatment (20 mM solution). 

### 2.7. Statistical Analysis 

All samples were analyzed in triplicate (*n* = 3) and the outcomes were reported as the mean ± standard deviation (SD). The data were statistically analyzed by Student’s t-test using Microsoft Office Excel computer software and by one-way analysis of variance (ANOVA) with post hoc Dunnett’s test using SigmaPlot 11.0 computer software. The difference showing a p level of 0.05 or lower was considered statistically significant. 

## 3. Results and Discussion 

### 3.1. Fitting the Experimental Data with the Models

The matrix of the experimental design containing the 17 formulations generated by the software and the results achieved after performing all the experimental runs are summarized in Table 3.

For data fitting, the partial least squares (PLS) method was employed and several statistical parameters were further used to assess the fitting results. The coefficient of correlation (*R*^2^) represents the variation of the response explained by the selected model, or goodness of fit, while the Q^2^ indicates the model capacity to be predictive. Furthermore, ANOVA test was employed to assess the experimental model validity, significance and lack of fit. The model proved good predictive ability, as shown by Q^2^ > 0.7, and a good correlation between the predicted and observed values was found, as suggested by *R*^2^ > 0.9 (Table 4). Differences of less than 0.2–0.3 between *R*^2^ and Q^2^ indicate a high predictive power of a good model. Models proved adequate validity (>0.4) corroborated with a reduced lack of fit for each evaluated response. The reproducibility values were >0.95, which means that the replicates generated similar responses by working under identical experimental conditions, thus making the experimental setup adequate for the purpose of the study. The results of ANOVA test showed a significant influence of the evaluated factors over TPC, TFC, CTC, and AA-TEAC, with *p*-value for the regression <0.001 (Table 4). Considering the results shown in Table 4, the fitting models were found to be appropriate to describe the experimental data, as the values for the lack of fit were not significant in extent with the pure error. The regression equation coefficients for the responses are presented in Table S4.

The influence of the extraction conditions (factors) on the quantified individual compound levels (responses) was studied using the second experimental design during the optimization step (Table 2). The factors were the same as in the screening step and the responses were the levels of the 14 bioactive compounds (polyphenols and sterols) (Table 5). Moreover, the samples were hydrolyzed and the results of the recovery for the main bioactive compounds were compared to those obtained from non-hydrolyzed samples (Table S5). The statistical parameters *R*^2^, Q^2^, regression, lack of fit and pure error were determined for fitting the experimental data with the experimental design. The selected model presented good quality, with *R*^2^ values between 0.75 and 0.94 and Q^2^ results in the range of 0.47–0.84. For all the evaluated responses, the results of ANOVA test for the model had statistical significance (*p* < 0.05) and lack of fit *p*-values in the range of 0.064–0.91 (Table S6). Considering the values obtained for these statistical parameters, the experimental data for the bioactive compounds were adequately described by the fitting models, with a quadratic model statistically significant and a low lack of fit. The evaluated responses were significantly influenced by the chosen factors. The regression equation coefficients for all the bioactive compounds determined in HI extracts are shown in Table S7. 

### 3.2. The Influence of Experimental Conditions on TPC, TFC, CTC, and AA-TEAC

As it can be noticed from the results for TPC, TFC, CTC, and AA-TEAC (Table 3), the responses were influenced by the factors used in the experimental design.

**Figure 1:**
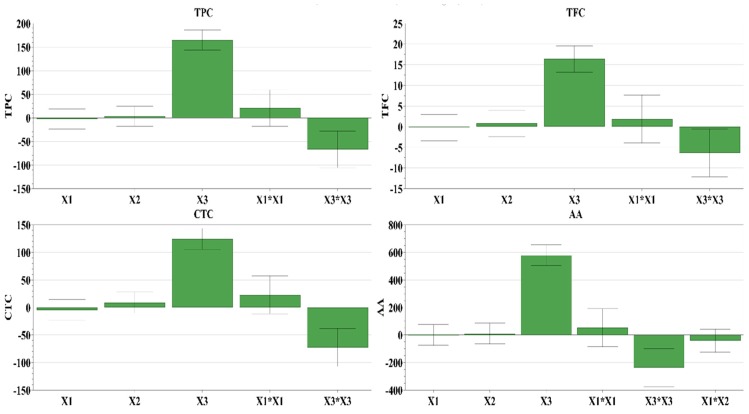
Influence of working conditions on total phenolic content (**TPC**), total flavonoid content (**TFC**), condensed tannin content (**CTC**), and antioxidant activity (**AA**) by TEAC assay of hazelnut involucre extracts, presented as scaled and centered coefficient plots. X_1_, stirring time (min); X_2_, pH; X_3_, water in solvent (% *v/v*).

**Figure 2:**
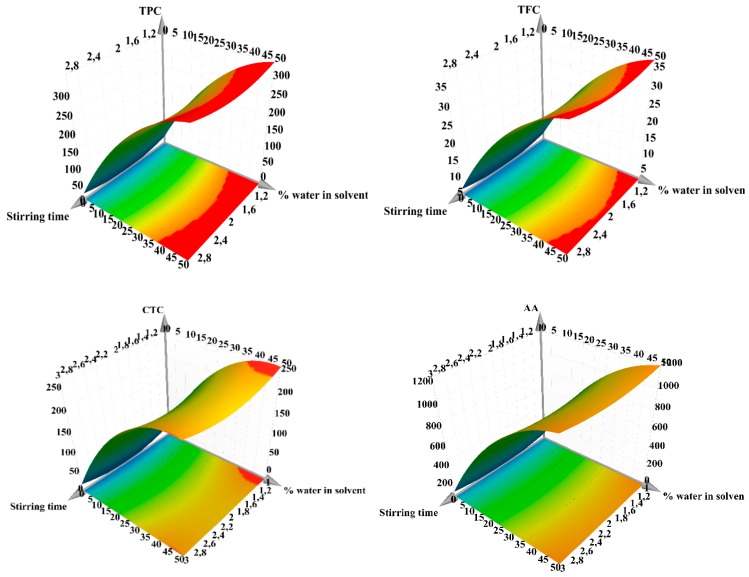
Response surface for predicting the recovery yield for total phenolic content (**TPC**), total flavonoid content (**TFC**), condensed tannin content (**CTC**,) and antioxidant activity (**AA**) by TEAC assay for hazelnut involucre extracts with regard to: X_1_, stirring time (min); X_3_, water in solvent (% *v/v*); X_2_, pH = 5. The regions in red represent the domains of working conditions assuring the maximum extraction yield for the evaluated bioactive compounds.

The stirring time was in the range of 1–3 min, the pH varied from acidic to neutral (3–5–7), and the two solvents used were water and acetone mixed in various proportions (0%–25%–50% water in acetone). The best extraction yields were obtained when using binary-solvent systems, while mono-solvent systems displayed a lower extraction power. These findings confirm the rational choice of the solvent mixture based on the previously reported determination of bioactive compounds in a study that aimed to evaluate the recovery efficiency from walnut septum extracts [15]. For a higher extraction yield, the best solvent mixture is between a polar, protic solvent (water) with a polar, relatively acidic, aprotic solvent (acetone). The best results for TPC, TFC, CTC, and AA-TEAC were obtained for pH 3 and 50% water in acetone, proving a positive relationship between the content of bioactive compounds and the antioxidant activity. The influences of working conditions on TPC, TFC, CTC, and AA-TEAC as scaled and centered coefficient plots are depicted in Figure 1, while the response surfaces for predicting the extraction efficiency for the aforementioned parameters are shown in Figure 2. From these figures, it can be observed that the factor with the highest positive impact on the recovery of bioactive compounds is the amount of water in the mixture solvent, a higher amount of water being associated with an increased extraction yield. In addition, the water amount proved to have a statistically significant influence upon TPC, TFC, CTC, AA-TEAC, and bioactive compound recovery (see Tables S4 and S7). The optimal extraction conditions for TPC, TFC, CTC, and AA-TEAC, generated by the software used, are given in Table 6.

### 3.3. Quantitative Determinations of Total Bioactive Compounds

As expected from previous studies [28], better extraction results of phenolic compounds found in HI, and responsible for the antioxidant activity and human health benefits, were obtained using a binary-solvent mixture of water and acetone. 

#### 3.3.1. Total Phenolic Content 

A significant difference in TPC values was noticed when an equal mixture of water and acetone was used compared to pure acetone. The value for the richest phenolic compound extract was 377.43 mg GAE/g dw HI when aqueous acetone solvent was used (N6, run order 7, Table 3). Because no similar data were found in the literature, where TPC was expressed using another phenolic compound reference (CE vs. GAE), we could not effectively compare the results. Thus, in the study of Alasalvar et al. [29], the TPC in hazelnut green leafy cover extracted with an aqueous 80% acetone or 80% ethanol were 201 or 156 mg catechin equivalent (CE)/g extract, respectively. The TPC in hazelnut green leafy cover obtained by Shahidi et al. [14], using a 80:20 (*v/v*) ethanol/water mixture as extraction solvent, was 127.3 mg CE/g extract, while the TPC for hazelnut skin, hard shell, tree leaf, and kernel were 577.7, 214.1, 134.7, and 13.7 mg CE/g extract, respectively. As seen in another study [30], TPC in tree nut by-products is several times higher compared to nut kernels. The quantification of TPC in different hazelnut kernel extracts exposed values in the range of 11.17–14.77 mg GAE/g [31] and 10.21–23.28 mg GAE/g [32]. 

Esposito et al. [33] detected quantities of 193.8 mg GAE/g methanol hazelnut shell extract for TPC, while Masullo et al. [34], for the same by-product, noticed that TPC was 340.44 μg GAE/mg extract, corresponding to 340.44 mg GAE/g. 

#### 3.3.2. Total Flavonoid Content 

Flavonoids exhibit both in vitro and in vivo biological activities and may elicit health benefits including cardiovascular protection [35]. In this study, the highest value for the TFC was 43.10 ± 1.59 mg QE/g dw involucre (N6, run order 7, Table 3). As in the case of phenolics, the best values for total flavonoids were obtained at equal volumes of water and acetone in the solvent mixture. 

To the best of our knowledge, there is no research regarding the flavonoid content in HI, therefore TFC values for nuts and other by-products were used as a comparison. The highest flavonoid content in walnut septum was 9.76 ± 0.23 mg QE/g [15], while hazelnuts presented TFC values between 0.09 ± 0.01 and 0.36 ± 0.07 mg rutin equivalent/g extract [36]. 

#### 3.3.3. Condensed Tannin Content 

Condensed tannins or proanthocyanidins had revealed positive effects on neurogenesis, cognitive improvement, and prevention of neuron death in neurodegenerative diseases such as Alzheimer’s disease [37].

In our study, the best value for CTC was 280.69 ± 7.85 mg CE/g dw HI, using as solvent 50% aqueous acetone solution (N6, run order 7, Table 3). Kim et al. [38] noticed values of 4.91 mg proanthocyanidins per gram of hazelnuts, while Lainas et al. [39] detected higher amounts of condensed tannins, 31.30 mg CE/g natural hazelnuts and 36.70 mg CE/g roasted hazelnut skin. The quantities for CTC obtained by Alasalvar et al. [29] were 385 and 542 mg CE/g extract from green leafy cover extracted with 80% ethanol in water and 80% acetone in water, respectively. The variations observed between the CTC results are due to different extraction conditions performed on various herbal matrices. The results of CTC found in the current study are in line with those obtained by Alasalvar et al. [29] on the same matrix. 

### 3.4. Determination of Antioxidant Activity

The antioxidant capacity of phenolic compounds depends basically on the number and position of the substituents on the aromatic ring [12]. However, because of their reduced bioavailability, due to extensive phase 2 metabolism, polyphenols might function more as up-regulators of the antioxidant activity than as direct antioxidants [40]. 

#### 3.4.1. TEAC Assay 

The antioxidant activity against the stable synthetic ABTS radical cation of different HI extracts is depicted in Table 3. This analysis is based on electron transfer reactions in order to assess the radical scavenging activity of several compounds. The highest values for AA were for 50% aqueous acetone extracts, with the strongest activity of 1296.51 ± 19.25 mg TE/g dw involucre (5.18 ± 0.08 mmol TE/g dw involucre). The AA for green leafy cover noticed by Shahidi et al. [14] was 117 µmol TE/g extract after extraction with 80% ethanol, while the values for hazelnut kernel, skin, hard shell, and tree leaf were between 29 and 148 µmol TE/g extract. Alasalvar et al. [29] reported AA values of 1.29 and 1.14 mmol TE/g extract obtained from green leafy cover using 80% acetone and 80% ethanol, respectively. In another source, the antioxidant activities were in the range of 3063–3573 µmol TE/100 g dw natural hazelnuts [41]. The AA of hazelnut by-product extracts was 4–5-fold greater than that of kernel extracts, meaning that hazelnut by-product extracts compared to hazelnut kernel extract would have a stronger antioxidant activity.

#### 3.4.2. DPPH Radical Scavenging Activity 

The DPPH radical scavenging assay was applied to measure the capability of HI to deactivate and scavenge this stable free radical. Antioxidant molecules can reduce DPPH free radicals, through hydrogen atom-donating capacity, and the absorbance will decrease faster if the antioxidant potential of the extract is more powerful [42].

In our study, the in vitro DPPH radical scavenging activity was 292.23 mg TE/g involucre extract obtained using 50% aqueous acetone solvent, at pH 3, and 3 min stirring time. The reported DPPH values on related matrices were between 84.9% and 93.6%, and 8.1% and 41.1% for natural hazelnuts and roasted hazelnuts, respectively [32]. In the study of Oliveira et al. [43], the EC_50_ values for hazelnut leaves obtained for DPPH radical scavenging were <0.3 mg/mL, while Masullo et al. [34] found the highest DPPH scavenging activity value (EC_50_ = 54.3 μg/mL) for hazelnut shells corresponding to the highest TPC value. Similarly, Yuan et al. [44] found a value of around 11 mg GAE/g for the richest TPC hazelnut shell extract. As there were no data regarding the DPPH assay for HI, a comparison with the results of other studies was not possible.

#### 3.4.3. FRAP Assay 

The FRAP assay, a fast and sensitive way for measuring the antioxidant capacity in samples via the reduction of ferric iron (Fe^3+^) to ferrous iron (Fe^2+^) by the antioxidants present in the samples, is a significant indication of the antioxidant activity. 

In our analysis, the reducing power for the richest polyphenolic HI extract achieved using water/acetone (1:1) at pH 3 and stirring time 3 min was 350.52 mg TE/g involucre extract. FRAP data for related matrices were 12.0 mg GAE/g hazelnut shell [44] and 400.97 mg TE/g walnut septum extract [15].

Our findings are in agreement with previous studies, which show a positive correlation between phenolic compounds and the antioxidant activity [45,46]. 

### 3.5. The Influence of Experimental Conditions on Individual Bioactive Compounds 

The individual polyphenols and phytosterols that were identified and quantified by LC-MS and LC-MS/MS in HI extracts are presented in Table 5. For epicatechin (Y_1_), catechin (Y_2_), syringic acid (Y_3_), gallic acid (Y_4_), protocatechuic acid (Y_5_), and vanillic acid (Y_6_) (Figure S1), the highest extraction yield was obtained for 2 min of stirring, at pH of 7, in a mixture of acetone/water (3:1). For *p*-coumaric acid (Y_7_) and ferulic acid (Y_8_) (Figure S2), the best recovery was attained for 1 min stirring time, pH 5, solvent mixture of acetone/water (3:1), while for hyperoside (Y_9_), isoquercitrin (Y_10_), and quercitrin (Y_11_) (Figure S3), the highest extraction power was observed for 3 min of stirring, at pH 3, in a mixture of solvents acetone/water in equal proportions (1:1). As far as stigmasterol and beta-sitosterol are concerned, their best recovery was obtained after 2 min of stirring the HI extracts in a mixture of solvents acetone/water (3:1) at pH 5, while the extraction of campesterol was the highest when stirring the sample for 3 min, at pH 5, in a mixture of acetone/water (3:1) (Table 5) (Figure S4). 

**Figure 3:**
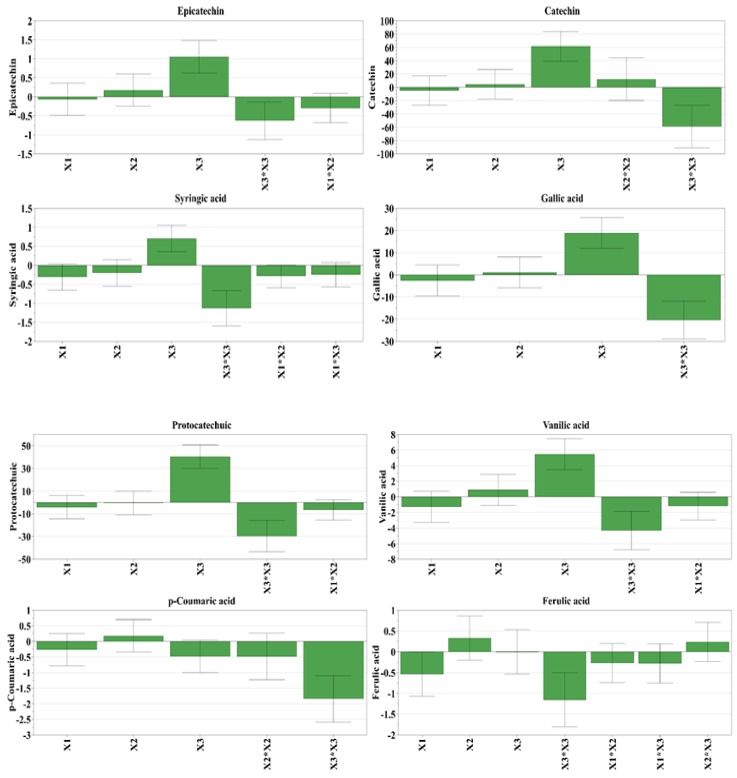

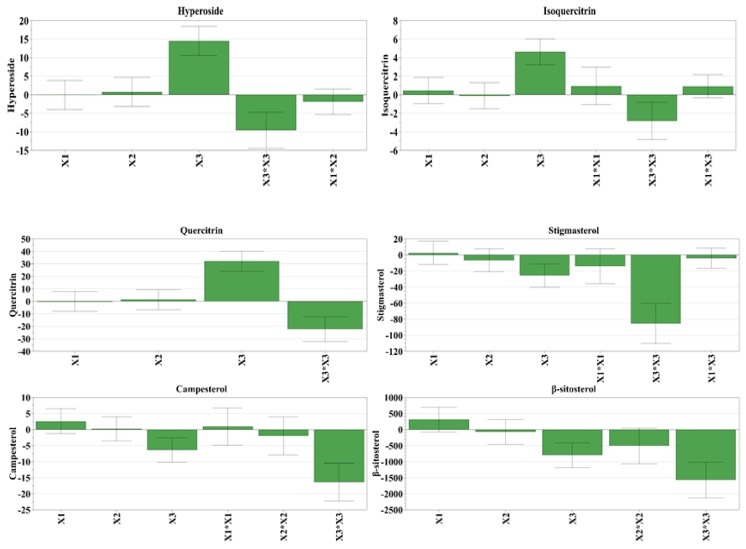
Influence of working conditions on the bioactive compound recovery from hazelnut involucre extracts, depicted as scaled and centered coefficient plots. X_1_, stirring time (min); X_2_, pH; X_3_, water in solvent (% *v/v*).

**Figure 4:**
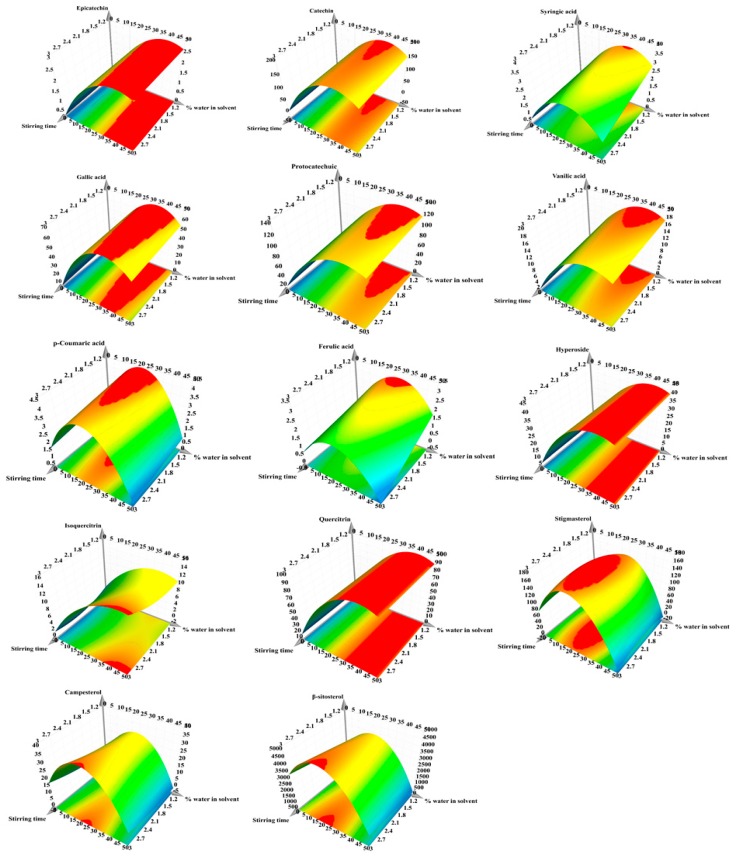
Response surface for prediction of bioactive compound recovery from hazelnut involucre extracts with respect to: X_1_, stirring time (min); X_2_, pH; X_3_, water in solvent (% *v/v*). The regions in red represent the domains of working conditions assuring the maximum extraction yield for the evaluated bioactive compounds.

From the 11 depicted polyphenols, catechin and protocatechuic acid were found in the highest quantity, whereas from the three revealed phytosterols, beta-sitosterol presented the highest amount. The influence of the working conditions on the evaluated bioactive compound recovery from HI extracts is depicted in Figure 3. Furthermore, for predicting the recovery of bioactive compounds considering the working conditions, the response surfaces were generated and presented in Figure 4. By analyzing the two figures, it can be concluded that the amount of water in the solvent mixture exhibited the highest impact on the recovery of bioactive compounds, followed by the stirring time. The pH displayed a minimum impact on the extraction yield.

We consider this information especially valuable because it presents the optimal experimental conditions for obtaining the maximum extraction yield.

#### 3.5.1. Quantification of Individual Polyphenols

From the 18 phenolic compounds analyzed by the validated LC-MS method, only five polyphenols (*p*-coumaric acid, ferulic acid, hyperoside, isoquercitrin, quercitrin) were quantified in the HI extracts. Using the LC-MS method II, all six polyphenols (epicatechin, catechin, syringic acid, gallic acid, protocatechuic acid, and vanillic acid) were quantified in the HI extracts. For this method, the coefficient of linearity (*R*^2^) was in the range of 0.9922–0.9997 and the accuracy bias was ≤15% [15].

The highest values for epicatechin and catechin were 3.73 and 243.02 μg/g dw HI, respectively (Table 5). Montella et al. [47] attained higher amounts of epicatechin and catechin, 342 and 2500 μg/g hazelnut skin extracts, a by-product richer in polyphenols. 

The maximum amounts for syringic acid, gallic acid, protocatechuic acid, and vanillic acid were 5.53, 91.93, 227.37, and 25.41 μg/g dw HI, respectively. In other matrices, Jakopic et al. [8] found quantities of 0.52 μg gallic acid and 2.92 μg protocatechuic acid per gram of hazelnut kernels, while Montella et al. [47], for the same hydroxybenzoic acids, gallic and protocatechuic acids, obtained concentration of 62.1 and 21.1 μg/g hazelnut skin extracts, respectively. 

The best quantities for two cinnamic acid derivatives, *p*-coumaric, and ferulic acids were 6.58 and 3.97 μg/g dw HI, respectively (Table 5). For the same two acids, Shahidi et al. [14] obtained values of 1662 and 327 μg/g green leafy cover extracts, respectively. Again, it is worth stating that many factors, including cultivar type, location, agricultural practices or growing conditions, degree of ripeness, storage conditions, and industrial processing, can affect the chemical composition of tree nut kernels and their by-products [11].

The highest quantities for hyperoside, quercitrin, and isoquercitrin were 51.72, 17.74, and 114.26 μg/g dw HI, respectively. 

The 17 hazelnut involucre extracts were hydrolyzed (as previously mentioned) and further analyzed by LC-MS for quantification of the main polyphenolic compounds (epicatechin, catechin, syringic acid, gallic acid, protocatechuic acid, vanillic acid). When comparing the non-hydrolyzed to the hydrolyzed samples (Table S5), the evaluated bio compounds registered a downward trend. This could be explained by the degradation of these polyphenols during the hydrolysis process, which was more significant than their release from the biological matrix or polymeric compounds.

#### 3.5.2. Quantification of Phytosterols

The three phytosterols (stigmasterol, campesterol, beta-sitosterol) found in HI extracts were quantified on the basis of their peak areas and evaluation with a calibration curve of the corresponding standards [25]. The richest extract in campesterol was 45.04 μg/g dw HI, while the highest quantities of stigmasterol and beta-sitosterol were 197.30 and 5305.01 μg/g dw HI extracts, respectively (Table 5). 

Phytosterols are known for the decreasing effect against total cholesterol and LDL-cholesterol, important risk factors in cardiovascular diseases [48]. Moreover, the consumption of plant sterols has been shown to increase the antioxidant activity [49]. Our study found quantities of phytosterols in HI which are in line with those in nuts [50] and proved that this by-product can be a source of natural sterols with several health benefits.

### 3.6. Enzyme Inhibitory Activities

#### 3.6.1. Selection of the Optimal HI Extract 

Polyphenols are bioactive compounds with potent antioxidant activity exercised by several mechanisms. In brief, they can act as direct and/or indirect antioxidants modulating many important cellular signaling pathways, such as: The nuclear factor erythroid 2-related factor 2/electrophile-responsive elements (Nrf2/EpRE) (involved in cell protection and detoxification) and the nuclear factor kappa B (NF-κB) (involved in the induction of pro-inflammatory processes). Polyphenols protect cells against free radicals and ROS, inhibiting mutagenesis and carcinogenesis by promoting apoptosis and autophagy in tumor cells. Moreover, they are epigenetic modulators, having real anticancer potential demonstrated in many in vitro and in vivo assays [5]. At the molecular level, extracts rich in polyphenols obtained from tree nut skin or by-products [15] showed inhibitory activity on key enzymes related to pathological conditions including type 2 diabetes mellitus (T2DM), obesity or skin hyperpigmentation [5].

Based on the experimental findings from the screening and optimization steps, the HI extract with the highest content in total polyphenols also displayed the best antioxidant capacity by TEAC assay (N6, run order 7, Table 3). Besides, this extract is the richest in hyperoside, isoquercitrin, and quercitrin (Table 5), flavonoids which are powerful antioxidants [15]. The extract was further evaluated for other biological activities: The antioxidant activities by two other assays, DPPH and FRAP (results presented above), the tyrosinase and α-glucosidase inhibitory activities, and the cytotoxic and antioxidant activities on tumor and normal cell lines.

#### 3.6.2. Tyrosinase Inhibitory Activity 

Tyrosinase is a key enzyme, which catalyzes melanin production, primarily responsible for the pigmentation of human skin, hair, and eyes. It also catalyzes the synthesis of neuromelanin in the human brain, and overproduction of neuromelanin is linked with neuronal damage and neurodegeneration in Parkinson’s disease and Huntington’s diseases. Tyrosinase was also associated with vegetable and fruit browning, which can lead to rapid postharvest degradation [51]. Tyrosinase inhibition may prevent skin hyperpigmentation and neurodegeneration. Therefore, tyrosinase downregulation, through natural inhibitors of this crucial enzyme, specifically targets melanogenesis in the cell with no side effects.

In our study, the tyrosinase inhibitory activity of 50% aqueous acetone HI extract was 165.17 ± 1.88 mg KAE/g. To the best of our knowledge, there were no previous studies of tyrosinase inhibitory activity of HI, thus no direct comparison could be made. Other plant matrices offered lower results, 129.98 ± 3.03 mg KAE per gram of walnut septum [15] and 2.28 ± 0.01 mg KAE/g of hazelnut extract [36]. Thus, HI may be an alternative natural source as a tyrosinase inhibitor in preventing hyperpigmentation, economical and convenient for the cosmetic and food industry. 

#### 3.6.3. α-Glucosidase Inhibitory Activity

Delayed glucose absorption and reducing postprandial blood glucose levels are antidiabetic strategies. The inhibition of the activity of α-glucosidase, an enzyme located on the epithelium of the small intestine, and the slowing of starch metabolism can be part of dietary therapy in the treatment of diabetes. Several plant extracts, rich in phenolic compounds and having structural features which may block the active sites of the enzyme, were shown to inhibit intestinal α-glucosidase [52]. Yeast α-glucosidase assay can be a fast and inexpensive method to screen for potential α-glucosidase inhibitors [27]. 

In our study, the IC_50_ for the 50% aqueous acetone HI extract was 0.10 mg/mL, which was much lower compared to acarbose, at 0.80 mg/mL, revealing HI extract as a more potent α-glucosidase inhibitor than acarbose, the anti-diabetic drug used in the treatment of T2DM. The enzyme inhibitory value for hazelnut was 7.57 ± 0.01 mmol acarbose equivalents per gram of extract [36].

It is possible that the enzymatic inducing effect of HI extract on both tyrosinase and α-glucosidase may be induced not only by the content of polyphenols but also by other compounds present in the extract. However, further research is needed to complete the phytochemical profile of this by-product and for the elucidation of the mechanisms of action.

### 3.7. Biological Activities of HI Extract on Cell Lines 

#### 3.7.1. Viability Assay

**Figure 5:**
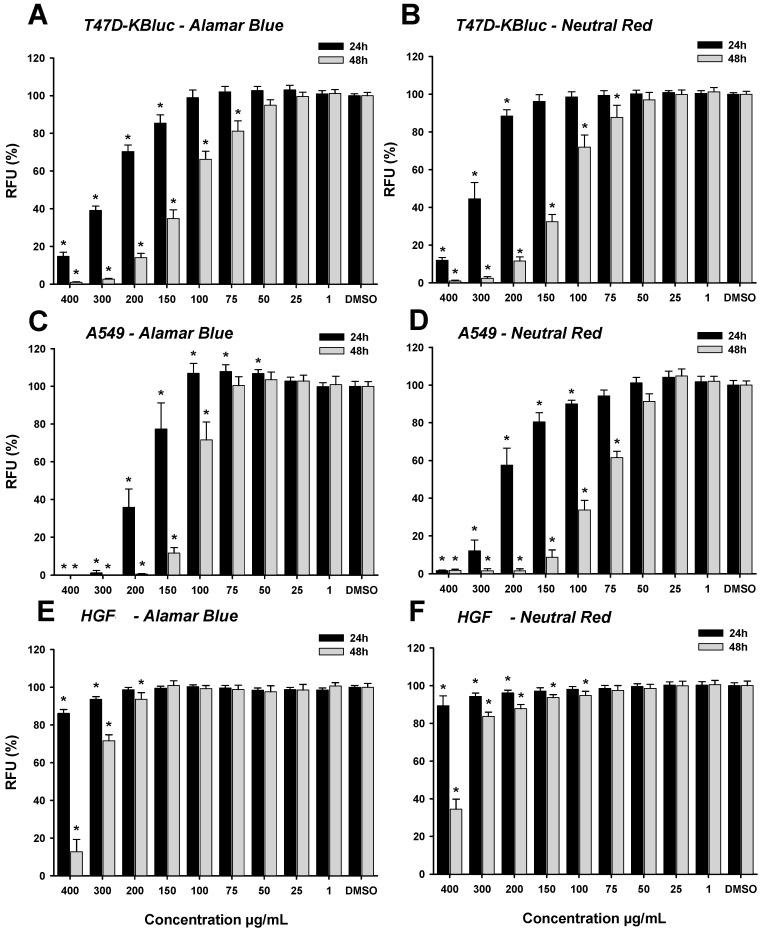
Cytotoxic effect of the HI extract observed using Alamar Blue assay on T47D-KBluc (**A**), A549 (**C**), and HGF (**E**) and using Neutral Red assay on T47D-KBluc (**B**), A549 (**D**), and HGF (**F**). The results are expressed as relative means ± standard deviations (six technical replicates for each of the three biological replicates) where the negative control (DMSO 0.2%) is 100%. (*) indicates significant differences compared to negative control (ANOVA + Dunnett’s; *p* < 0.05).

Exposure of T47D-KBluc and A549 cell lines to the polyphenolic richest HI extract resulted in a dose-dependent decrease in cellular viability, with a comparable susceptibility to the extract observed for both cancerous cell lines in both assays. The calculated IC_50_, defined here as the concentration of the HI extract that induces the cellular death of 50% of the total viable cells, at 24 h and 48 h after exposure of the cell lines, based on the data from Alamar Blue and Neutral Red assays are presented in Table 7. In comparison with the T47D-KBluc cell line, the A549 cell line appeared to be more sensitive to the toxic effects of the HI extracts. In addition, when the A549 cells were exposed for 24 h to the HI extract, a hormetic effect was observed at intermediate concentrations in the case of Alamar Blue assay (Figure 5). The toxicity of the extract was more prominent after 48 h exposure for all the cell lines tested, with IC_50_ reaching almost half of the values observed at 24 h post-exposure in the case of A549 and T47D-KBluc (Table 7). Contrary to the results observed when using cancerous cell lines, exposure of the normal HGF cells to the HI extract resulted in a mild cytotoxic effect, observed only at the highest tested doses.

Similarly to the results reported by Gallego et al. [53], the present results corroborate to the idea that extracts from different parts of hazelnut plant can exert a cytotoxic effect on cancerous cell lines. A direct comparison between our study and the previously mentioned study is not possible as only a limited number of higher concentrations were tested in the latter study, while the extracts were obtained from stems and leaves of hazel trees [53]. In agreement with other studies in the literature, due to the presence of active compounds such as polyphenols, flavonoids, or other plant-specific compounds such as taxanes present in *Corylus avellana* L., plant-derived extracts can inhibit the growth of breast, gastric, and pulmonary tumor-derived cell lines [54,55,56,57]. 

#### 3.7.2. DCFH-DA Assay

**Figure 6:**
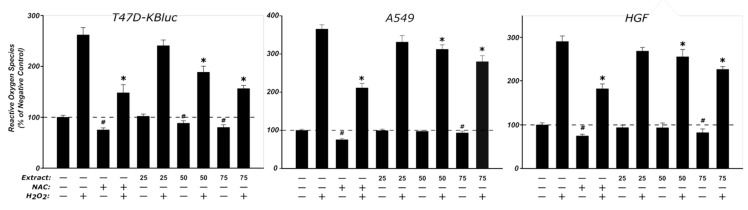
Antioxidant effect of the HI extract evaluated using DCFH-DA assay on T47D-KBluc, A549, and HGF. The cellular model was pre-exposed to the extract (25, 50, and 75 μg/mL) or NAC (20 mM) for 24 h, and further incubated with 50 μM DCFH-DA. The antioxidant effect of the HI extract was assessed after 2 h from oxidative stress induced by exposure to 250 μM H_2_O_2_. The results are expressed as relative means ± standard deviations (six technical replicates for each of the three biological replicates) where the negative control (DMSO 0.2%) is 100%. (*) indicates significant differences compared to H_2_O_2_ exposure alone; (#) indicates significant differences compared to negative control (ANOVA + Dunnett’s; *p* < 0.05).

In order to evaluate a potential antioxidant effect of the HI extract, three concentrations (25, 50, 75 µg/mL) that did not affect the cellular viability were selected and further tested on the two cancerous cell lines and on the normal HGF cells (Figure 6). The treatment with the HI extract alone modified the oxidative status of all three cell lines, with a statistical decrease of ROS being observed at the highest tested doses. Similar to the HI extract, NAC treatment alone led to a decrease in the basal oxidative status compared to the negative control. Exposure to H_2_O_2_ alone resulted in an approximately threefold increase of the signal when compared to the negative control, while pre-exposure to the antioxidant NAC decreased the ROS generation by approximately 50%. Pre-incubation with HI extract decreased the ROS in a dose-dependent manner, reaching statistical significance at the two highest doses of 50 and 75 µg/mL. In the case of T47D-KBluc cells, at the highest dose, the reduction of oxidative stress was almost equal to the one exerted by NAC.

The obtained results are in agreement with the previously detailed antioxidant activity observed in the non-cellular assays, but due to insufficient data present in literature, no direct comparison was possible.

## 4. Conclusions

This study characterized the phytochemical profile of hazelnut (*Corylus avellana* L.) involucre and described the optimal experimental conditions for maximizing the extraction efficiency of biologically active compounds from this tree nut by-product. We aimed to obtain hazelnut involucre extracts with high contents of phenolics, strong antioxidant capacity, and biological potential based on experimental designs. Besides, the phytochemical profile of the extracts using LC-MS methods was assayed. In order to determine the ideal extraction conditions, several parameters (stirring time, pH, solvent) were coupled with chemical analysis and statistical tools. From the eleven polyphenols (epicatechin, catechin, syringic acid, gallic acid, protocatechuic acid, vanillic acid, *p*-coumaric acid, ferulic acid, hyperoside, isoquercitrin, quercitrin) and three phytosterols (stigmasterol, campesterol, beta-sitosterol) determined and quantified, catechin (up to 0.243 mg/g dw HI), protocatechuic acid (up to 0.227 mg/g dw HI), and beta-sitosterol (up to 5.305 mg/g dw HI) were found in the highest amount. From the extraction conditions, the amount of water in the solvent proved to have a statistically significant influence upon all evaluated responses.

The best extraction conditions to attain the richest extract in total phenols were accomplished using turbo-extraction by Ultra-Turrax at stirring time 3 min, pH 3, and 50% acetone in water (*v/v*) as extraction solvent, while the best extraction conditions for sterols were 75% acetone in water (*v/v*), pH 5, and stirring time 2 or 3 min. The richest extract in polyphenols presented strong in vitro antioxidant activity in classical tests (TEAC, DPPH, FRAP) as well as in two cancer cell line assays (T47D-KBluc and A549). Cytotoxic and antioxidant effects of HI extract were more intense on these cancer cells than on a normal cell line (HGF). These results are promising and justify further research to characterize HI as a valuable source of bioactive compounds and to assay its anticancer potential. Moreover, the richest polyphenol HI extract demonstrated good enzyme inhibitory potential on tyrosinase (165.17 ± 1.88 mg KAE/g) and α-glucosidase (eight times stronger inhibition than acarbose), two key enzymes involved in age-related diseases. Phytosterols, compounds with proven cardioprotective effects and antioxidant capacity, are other valuable molecules found in HI.

The results of our study successfully accomplished the proposed objectives and justify future scientific investigations, including a comprehensive identification of individual polyphenols. Moreover, the content in bioactive compounds, correlated with good results for antioxidant and enzyme inhibitory activities, warrants more research in order to understand the bioavailability of specific molecules and to reveal their potential mechanisms of action for future use of hazelnut involucre in the cosmetic industry, in addition to food and pharmaceutical production.

## Figures and Tables

**Table 1:** Independent and dependent variables of the experimental design used in the screening step.

Variables	Level		
	−1	0	1
*Independent variables (factors)*			
Stirring time (min) (X_1_)	1	2	3
pH (X_2_)	3	5	7
Water in solvent (%, *v/v*) (X_3_)	0	25	50
*Dependent variables (responses)*			
Total phenolic content (TPC, mg GAE/g dw ^1^) (Y_1_)			
Total flavonoid content (TFC, mg QE/g dw ^2^) (Y_2_)			
Condensed tannin content (CTC, mg CE/g dw ^3^) (Y_3_)			
Antioxidant activity (AA, mg TE/g dw ^4^) (Y_4_)			

^1^—mg GAE/g dw = gallic acid equivalents per dry weight of hazelnut involucre, ^2^—mg QE/g dw = quercetin equivalents per dry weight of hazelnut involucre, ^3^—mg CE/g dw = catechin equivalents per dry weight of hazelnut involucre, ^4^—mg TE/g dw = Trolox equivalents per dry weight of hazelnut involucre.

**Table 2:** Independent and dependent variable of experimental design used in the optimization step.

Variables	Level		
	−1	0	1
*Independent variables (factors)*			
Stirring time (min) (X_1_)	1	2	3
pH (X_2_)	3	5	7
Water in solvent (%, *v/v*) (X_3_)	0	25	50
*Dependent variables (responses)*			
Epicatechin (μg/g dw) (Y_1_)			
Catechin (μg/g dw) (Y_2_)			
Syringic acid (μg/g dw) (Y_3_)			
Gallic acid (μg/g dw) (Y_4_)			
Protocatechuic acid (μg/g dw) (Y_5_)			
Vanillic acid (μg/g dw) (Y_6_)			
*p*-Coumaric acid (μg/g dw) (Y_7_)			
Ferulic acid (μg/g dw) (Y_8_)			
Hyperoside (μg/g dw) (Y_9_)			
Isoquercitrin (μg/g dw) (Y_10_)			
Quercitrin (μg/g dw) (Y_11_)			
Stigmasterol (μg/g dw) (Y_12_)			
Campesterol (μg/g dw) (Y_13_)			
Beta-sitosterol (μg/g dw) (Y_14_)			

dw—dry weight hazelnut involucre.

**Table 3:** Matrix of experimental design and results for total phenolic content, total flavonoid content, condensed tannin content, and antioxidant activity of hazelnut involucre extracts based on a factorial design during the screening step.

Sample Code	Run Order	Factorial Design with Coded Values			Determination (Experimental)			
		X_1_	X_2_	X_3_	Y_1_ (TPC)	Y_2_ (TFC)	Y_3_ (CTC)	Y_4_ (AA-TEAC)
N1	12	1	3	0	2.62 ± 0.35	2.23 ± 0.23	0.04 ± 0.02	9.86 ± 1.13
N2	3	3	3	0	4.88 ± 0.66	2.88 ± 0.10	0.26 ± 0.14	18.89 ± 1.62
N3	2	1	7	0	4.33 ± 0.41	3.64 ± 0.18	1.48 ± 0.85	16.01 ± 1.07
N4	14	3	7	0	2.59 ± 0.19	1.80 ± 0.09	0.57 ± 0.08	8.65 ± 0.53
N5	10	1	3	50	320.83 ± 24.42	32.12 ± 1.14	226.74 ± 3.69	1049.75 ± 25.43
N6	7	3	3	50	377.43 ± 26.74	43.10 ± 1.59	280.69 ± 7.85	1296.51 ± 19.25
N7	1	1	7	50	332.78 ± 19.62	39.82 ± 0.92	274.27 ± 14.73	1207.62 ± 30.23
N8	13	3	7	50	334.68 ± 16.78	37.32 ± 0.70	242.31 ± 1.06	1137.48 ± 47.46
N9	4	1	5	25	292.98 ± 9.49	34.47 ± 1.39	244.06 ± 4.03	1065.73 ± 55.15
N10	8	3	5	25	210.67 ± 5.46	25.04 ± 2.77	174.81 ± 3.02	885.31 ± 17.82
N11	9	2	3	25	188.82 ± 0.60	22.01 ± 1.02	150.18 ± 1.19	718.15 ± 21.35
N12	5	2	7	25	257.22 ± 8.50	28.06 ± 1.04	228.27 ± 2.33	823.02 ± 46.84
N13	16	2	5	0	15.50 ± 0.39	10.56 ± 0.51	2.19 ± 0.15	109.79 ± 26.74
N14	6	2	5	50	313.21 ± 8.00	32.56 ± 0.45	225.72 ± 1.99	1261.77 ± 180.09
N15	17	2	5	25	197.39 ± 16.29	25.53 ± 0.21	180.87 ± 4.21	760.24 ± 114.77
N16	11	2	5	25	180.95 ± 10.42	25.38 ± 1.43	174.78 ± 13.29	749.62 ± 97.78
N17	15	2	5	25	196.32 ± 15.63	25.30 ± 0.58	169.95 ± 2.18	714.15 ± 93.65

X_1_, stirring time; X_2_, pH; X_3_, water in solvent (%, *v/v*). TPC: Total phenolic content expressed as mg GAE/g dw = gallic acid equivalents per dry weight of hazelnut involucre; TFC: Total flavonoid content expressed as mg QE/g dw = quercetin equivalents per dry weight of hazelnut involucre; CTC: Condensed tannin content expressed as mg CE/g dw = catechin equivalents per dry weight of hazelnut involucre; AA-TEAC: Antioxidant activity by TEAC assay expressed as mg TE/g dw = Trolox equivalents per dry weight of hazelnut involucre. Data are shown as mean ± SD (standard deviation).

**Table 4:** Statistical parameters after data analysis and fit with factorial model.

Evaluated Response	Total Phenolic Content					Total Flavonoid Content					Condensed Tannin Content					Antioxidant Activity				
	(*R*^2^ = 0.96, Q^2^ = 0.91)					(*R*^2^ = 0.92, Q^2^ = 0.78)					(*R*^2^ = 0.95, Q^2^ = 0.88)					(*R*^2^ = 0.97, Q^2^ = 0.92)				
Reproducibility	0.95					0.98					0.97					0.99				
	SS	DF	MS	*F-v*	*p-v*	SS	DF	MS	*F-v*	*p-v*	SS	DF	MS	*F-v*	*p-v*	SS	DF	MS	*F-v*	*p-v*
Regression	286,363	5	57,272.6	61.46	0.001	2819.57	5	563.91	26.71	0.001	1728.27	5	345.65	46.74	0.001	5.26 × 10^5^	6	878,126	69.9	0.001
Lack of fit	8678.93	9	964.32	1.22	0.527	227.15	9	25.23	9.99	0.094	75.94	9	8.43	3.12	0.266	122,720	8	15,340	10.58	0.089
Pure error	1570.49	2	785.24			5.04	2	2.52			5.39	2	2.69			2899.26	2	1449.63		

*R*^2^: Coefficient of correlation/goodness of fit; Q^2^: Goodness of prediction; SS: Sum of squares; DF: Degrees of freedom; MS: Mean square; F-v: F-value, Fischer’s ratio; p-v: *p*-value, probability.

**Table 5:** Matrix of experimental design and results for bioactive compound recovery from hazelnut involucre extracts.

Sample Code	Run Order	Factorial Design with Coded Values			Determination (Experimental Results)													
		X_1_	X_2_	X_3_	Y_1_	Y_2_	Y_3_	Y_4_	Y_5_	Y_6_	Y_7_	Y_8_	Y_9_	Y_10_	Y_11_	Y_12_	Y_13_	Y_14_
N1	12	1	3	0	ND	ND	ND	ND	ND	ND	0.37	0.23	1.23	0.43	3.78	30.05	3.45	916.69
N2	3	3	3	0	0.16	13.62	0.54	5.29	21.73	3.10	0.94	0.54	2.69	1.13	8.36	61.06	5.75	2444.00
N3	2	1	7	0	0.11	10.28	0.41	3.78	14.55	2.44	0.70	0.44	3.09	1.01	7.32	30.05	2.32	1442.87
N4	14	3	7	0	ND	ND	ND	ND	ND	ND	0.81	0.49	2.04	0.80	6.51	36.02	5.88	1408.88
N5	10	1	3	50	1.48	155.09	2.65	55.39	103.98	15.78	ND	ND	31.61	9.21	76.21	ND	ND	ND
N6	7	3	3	50	3.61	201.95	2.58	63.59	131.15	16.61	ND	ND	51.72	17.74	114.26	ND	ND	ND
N7	1	1	7	50	3.17	158.06	2.77	53.14	124.58	21.10	ND	3.23	43.90	10.25	97.15	ND	ND	ND
N8	13	3	7	50	2.05	161.14	ND	42.87	103.52	12.29	ND	ND	42.26	13.29	94.52	ND	ND	ND
N9	4	1	5	25	3.48	216.97	3.33	69.07	140.91	18.71	6.58	3.97	50.99	15.71	112.05	ND	25.35	3145.78
N10	8	3	5	25	1.62	108.01	2.01	37.15	70.58	9.83	3.19	ND	30.93	9.10	71.32	195.28	45.04	5166.14
N11	9	2	3	25	1.65	150.77	2.63	53.23	97.26	14.51	2.74	2.49	31.04	8.64	71.34	185.63	31.38	3792.66
N12	5	2	7	25	3.73	243.03	5.53	91.93	227.37	25.41	4.41	3.30	36.23	10.31	85.39	145.24	24.79	3480.22
N13	16	2	5	0	0.27	19.98	0.87	11.83	33.32	4.71	2.14	1.51	5.89	ND	17.99	77.68	12.66	2843.16
N14	6	2	5	50	ND	78.05	2.41	44.25	100.40	13.61	ND	ND	29.52	8.60	67.21	ND	ND	ND
N15	17	2	5	25	2.41	172.79	3.39	54.21	119.09	14.81	3.14	2.15	2.44	9.57	85.62	141.49	28.96	3821.43
N16	11	2	5	25	1.98	159.99	2.65	61.39	103.95	12.48	3.91	2.89	36.71	10.82	80.39	197.31	31.49	5305.01
N17	15	2	5	25	1.89	186.77	3.78	57.64	116.71	15.31	3.61	2.65	39.23	11.98	88.73	178.96	22.76	3213.27

X_1_, stirring time (min); X_2_, pH; X_3_, water in solvent (%, *v/v*). Y_1_: Epicatechin; Y_2_: Catechin; Y_3_: Syringic acid; Y_4_: Gallic acid; Y_5_: Protocatechuic acid; Y_6_: Vanillic acid; Y_7_: *p*-Coumaric acid; Y_8_: Ferulic acid; Y_9_: Hyperoside; Y_10_: Isoquercitrin; Y_11_: Quercitrin; Y_12_: Stigmasterol; Y_13_: Campesterol; Y_14_: Beta-sitosterol. All responses are expressed as μg bioactive compound per gram of dry weight hazelnut involucre. ND—not determined.

**Table 6:** Optimum experimental conditions for improved recovery of TPC, TFC, CTC, AA-TEAC from hazelnut involucre extracts.

Parameters	TPC	TFC	CTC	AA-TEAC
**Stirring time (min)**	3	3	3	3
**pH**	3	3	3	3
**Water in solvent (%)**	50	50	50	50
**Predicted**	345.96	38.41	269.67	1253.09
**Determined**	370.42	41.97	279.30	1291.22
**Bias (%)**	7.07	9.26	3.57	3.04

TPC: Total phenolic content expressed as mg GAE/g dw = gallic acid equivalents per dry weight of hazelnut involucre; TFC: Total flavonoid content expressed as mg QE/g dw = quercetin equivalents per dry weight of hazelnut involucre; CTC: Condensed tannin content expressed as mg CE/g dw = catechin equivalents per dry weight of hazelnut involucre; AA-TEAC: Antioxidant activity by TEAC assay expressed as mg TE/g dw = Trolox equivalents per dry weight of hazelnut involucre.

**Table 7:** IC_50_ values (µg extract/mL) obtained by Alamar Blue and Neutral Red assays for T47D-KBluc, A549, and HGF at 24 h and 48 h post-exposure.

Cell Lines	24 h		48 h	
	Alamar Blue	Neutral Red	Alamar Blue	Neutral Red
**T47D-KBluc**	281.41 ± 22.7	294.56 ± 4.03	123.62 ± 1.97	125.042 ± 1.15
**A549**	180.28 ± 4.6	222.10 ± 15.05	112.53 ± 1.44	83.06 ± 1.33
**HGF**	˃400	˃400	˃300	˃300

T47D-KBluc: Human breast cancer; A549: Human lung adenocarcinoma; HGF: Human gingival fibroblasts.

## Supplementary Materials

The following are available online at https://www.mdpi.com/2076-3921/8/10/460/s1, Figure S1: The chromatograms for the individual polyphenolic compounds quantified by the LC-MS method II according to the retention time and m/z values in Supplementary Table S2, Figure S2: The chromatograms for the individual polyphenolic compounds quantified by an LC-MS method according to the retention time and m/z values in Supplementary Table S1, Figure S3: The chromatograms for the individual polyphenolic compounds quantified by an LC-MS method according to the retention time and m/z values in Supplementary Table S1, Figure S4: The chromatograms for phytosterols quantified by an LC-MS/MS method according to the retention time and m/z values in Supplementary Table S3; Table S1: Retention times and values of m/z for the individual polyphenolic compounds, Table S2: Retention times and values of m/z for the individual polyphenolic compounds quantified by LC-MS method II, Table S3: Retention times (RT) and specific ions of the phytosterols, Table S4: Regression equation coefficients for total bioactive compounds in hazelnut involucre extracts, Table S5: Quantitative evaluation of the recovery of main bioactive compounds in non-hydrolyzed and hydrolyzed samples of hazelnut involucre extracts, Table S6: Statistical parameters after data analyze and fit with factorial model for bioactive compounds in hazelnut involucre extracts, Table S7: Regression equation coefficients for individual bioactive compounds in hazelnut involucre extracts.
