# Supplementary material for: Enhanced Recovery of Antioxidant Compounds from Hazelnut (Corylus avellana L.) Involucre Based on Extraction Optimization: Phytochemical Profile and Biological Activities
Source: Antioxidants (Basel). 2019 Oct 8;8(10):460. doi: 10.3390/antiox8100460 (PMC6826866; doi:10.3390/antiox8100460)
Supplement: Supplementary file 1 [file antioxidants-08-00460-s001.pdf]

Supplementary Table S1. Retention times and values of m/z for the individual polyphenolic compounds.

**Phenolic compound**	***m/z***	**Retention time (min)**
Caftaric acid	311	3.5
Gentisic acid	179	3.5
Caffeic acid	179	5.6
Chlorogenic acid	353	5.6
p-Coumaric acid	163	9.5
Ferulic acid	193	12.8
Sinapic acid	223	15.0
Hyperoside	463	18.6
Isoquercitrin	463	19.6
Rutoside	609	20.7
Myricetin	317	21.1
Fisetin	285	22.9
Quercitrin	447	23.6
Quercetin	301	26.8
Patuletin	331	29.4
Luteolin	285	29.1
Kaempferol	285	32.5
Apigenin	279	33.1

Supplementary Table S2. Retention times and values of m/z for the individual polyphenolic compounds quantified by LC-MS method II.

**Compounds**	***m/z***	**Retention time (min)**
Epicatechin	289	9.0
Catechin	289	6.0
Syringic acid	197	8.4
Gallic acid	169	1.5
Protocatechuic acid	153	2.8
Vanillic acid	167	6.7

Supplementary Table S3. Retention times (RT) and specific ions of the phytosterols.

Phytosterol	RT (min)	M	M – H_2O + H^+
Ergosterol	3.2	396	379
Brassicasterol	3.9	398	381
Stigmasterol	4.9	412	395
Campesterol	4.9	400	383
β-Sitosterol	5.7	414	397

Supplementary Table S4. Regression equation coefficients for total bioactive compounds in hazelnut involucre extracts.

Effect	Responses			
	**Y_1 (Total Phenolic Content)**	**Y_2 (Total Flavonoid Content)**	**Y_3 (Condensed Tannin Content)**	**Y_4 (Antioxidant Activity)**
Constant	**217.984**	**25.159**	**17.627**	**769.490**
X_1 (stirring time)	-1.856	-0.169	-0.385	-2.943
X_2 (pH)	2.950	0.661	0.716	**100.698**
X_3 (water in solvent)	**131.450**	**13.046**	**10.009**	**551.116**
X_1 x X_1	6.249	0.526	0.726	81.923
X_3 x X_3	**-31.751**	-3.016	**-3.472**	-84.811
X_2 x X_3	**-**	**-**	-	**91.948**

For data in bold, p-value was <0.005, therefore statistically significant.

Supplementary Table S5. Quantitative evaluation of the recovery of main bioactive compounds in non-hydrolyzed and hydrolyzed samples of hazelnut involucre extracts.

Sample code/ Bioactive compound	Non-hydrolyzed samples						Hydrolyzed samples					
	Epicatechin	Catechin	Syringic acid	Gallic acid	Protocatechuic acid	Vanillic acid	Epicatechin	Catechin	Syringic acid	Gallic acid	Protocatechuic acid	Vanillic acid
N1	ND	ND	ND	ND	ND	ND	ND	ND	ND	ND	ND	ND
N2	0.161245	13.622	0.544025	5.29325	21.7316	3.10038	ND	ND	1.265 x 10^{-6}	3.085 x 10^{-5}	3.348 x 10^{-5}	7.899 x 10^{-6}
N3	0.109285	10.2814	0.406842	3.78198	14.5526	2.43934	ND	ND	ND	ND	ND	ND
N4	ND	ND	ND	ND	ND	ND	ND	ND	ND	ND	ND	ND
N5	1.48028	155.086	2.65383	55.3962	103.981	15.7851	ND	ND	ND	ND	ND	ND
N6	3.61175	201.95	2.57709	63.593	131.153	16.6086	ND	ND	ND	ND	ND	ND
N7	3.17511	158.064	2.77131	53.1407	124.579	21.1011	ND	1.980 x 10^{-5}	1.190x 10^{-5}	6.960 x 10^{-4}	2.991 x 10^{-4}	6.407 x 10^{-5}
N8	2.0507	161.137	ND	42.872	103.519	12.2973	ND	ND	ND	ND	ND	ND
N9	3.48134	216.972	3.32771	69.0716	140.899	18.7113	3.286 x 10^{-5}	5.835 x 10^{-5}	1.497 x 10^{-5}	10.279 x 10^{-4}	5.014 x 10^{-4}	0.00011248
N10	1.62016	108.011	2.00567	37.151	70.5825	9.83373	ND	1.816 x 10^{-5}	1.124 x 10^{-5}	9.872 x 10^{-4}	4.362 x 10^{-4}	0.000102659
N11	1.64843	150.773	2.62852	53.2252	97.2631	14.5142	ND	4.618 x 10^{-6}	1.491 x 10^{-5}	9.259 x 10^{-4}	4.104 x 10^{-4}	9.392 x 10^{-5}
N12	3.73195	243.025	5.53267	91.9302	227.373	25.4056	ND	ND	ND	ND	ND	ND
N13	0.272933	19.9771	0.873456	11.8345	33.3216	4.70599	ND	ND	ND	ND	ND	ND
N14	ND	78.0445	2.40167	44.2498	100.404	13.5972	2.694 x 10^{-5}	7.058 z 10^{-5}	9.818 x 10^{-6}	8.0096 x 10^{-4}	2.858 x 10^{-4}	7.127x 10^{-5}
N15	2.4124	172.79	3.39555	54.205	119.09	14.8084	1.885 x 10^{-5}	3.732 x 10^{-5}	1.011 x 10^{-5}	6.888 x 10^{-4}	3.343 x 10^{-4}	6.682 x 10^{-5}
N16	1.98295	159.987	2.64634	61.3946	103.949	12.4836	ND	ND	ND	ND	ND	ND
N17	1.89834	186.77	3.78313	57.6435	116.706	15.3084	ND	ND	ND	ND	ND	ND

All results are expressed as μg bioactive compound per gram of dry weight hazelnut involucre.

Supplementary Table S6. Statistical parameters after data analyze and fit with factorial model for bioactive compounds in hazelnut involucre extracts.

Quantifiable responses	Reproducibility	Source	Degrees of freedom	Sum of squares	Mean square	F value	*p* value
Epicatechin (Y_1) (R^2=0.79, Q^2=0.51)	0.95	Regression	5	21.2182	4.24364	7.83307	0.003
		Lack of fit	8	5.26564	0.658205	8.66356	0.108
		Pure error	2	0.151948	0.075974		
Catechin (Y_2) (R^2=0.83, Q^2=0.63)	0.97	Regression	5	91497.7	18299.5	11.1942	0.001
		Lack of fit	9	17623.3	1958.14	10.912	0.087
		Pure error	2	358.896	179.448		
Syringic acid (Y_3) (R^2=0.88, Q^2=0.59)	0.81	Regression	6	23.7157	3.95262	11.1702	0.001
		Lack of fit	7	2.51674	0.359534	1.07654	0.561
		Pure error	2	0.667942	0.333971		
Gallic acid (Y_4) (R^2=0.83, Q^2=0.73)	0.98	Regression	4	10194.3	2548.57	15.5436	0.001
		Lack of fit	10	1941.7	194.17	15.0162	0.064
		Pure error	2	25.8614	12.9307		
Protocatechuic acid (Y_5) (R^2=0.91, Q^2=0.71)	0.97	Regression	5	33137.3	6627.46	20.6287	0.001
		Lack of fit	8	3080.17	385.021	5.8091	0.155
		Pure error	2	132.558	66.2791		
Vanillic acid (Y_6) (R^2=0.83, Q^2=0.64)	0.95	Regression	5	741.904	148.381	11.246	0.001
		Lack of fit	9	140.59	15.6211	6.8744	0.133
		Pure error	2	4.54473	2.27237		
p-Coumaric acid (Y_7) (R^2=0.84, Q^2=0.68)	0.96	Regression	5	53.2525	10.6505	11.9547	0.001
		Lack of fit	9	9.50473	1.05608	7.15409	0.129
		Pure error	2	0.295239	0.147619		
Ferulic acid (Y_8) (R^2=0.75, Q^2=0.47)	0.92	Regression	7	24.823	3.54615	4.02297	0.028
		Lack of fit	7	7.64398	1.092	7.54937	0.122
		Pure error	2	0.289295	0.144647		
Hyperoside (Y_9) (R^2=0.88, Q^2=0.66)	0.96	Regression	5	4446.64	889.328	17.3971	0.001
		Lack of fit	9	538.734	59.8593	5.07763	0.175
		Pure error	2	23.5777	11.7888		
Isoquercitrin (Y_{10}) (R^2=0.87, Q^2=0.55)	0.95	Regression	6	427.541	71.2568	11.1563	0.001
		Lack of fit	8	60.9784	7.6223	5.26948	0.169
		Pure error	2	2.893	1.4465		
Quercitrin (Y_{11}) (R^2=0.89, Q^2=0.76)	0.98	Regression	4	21696.5	5424.12	25.0412	0.001
		Lack of fit	10	2563.78	256.378	14.4347	0.066
		Pure error	2	35.5223	17.7611		

Quantifiable responses	Reproducibility	Source	Degrees of freedom	Sum of squares	Mean square	F value	*p* value
Stigmasterol (Y_{12}) (R^2=0.94, Q^2=0.84)	0.87	Regression	6	90258.8	15043.1	24.7512	0.001
		Lack of fit	7	3851.44	550.206	0.679889	0.707
		Pure error	2	1618.52	809.259		
Campesterol (Y_{13}) (R^2=0.91, Q^2=0.65)	0.91	Regression	6	3009.65	501.609	14.5167	0.001
		Lack of fit	6	236.115	39.3525	1.95216	0.377
		Pure error	2	40.317	20.1585		
Beta-sitosterol (Y_{14}) (R^2=0.90, Q^2=0.74)	0.66	Regression	5	4.92551×10^{-7}	9.85102×10^{-6}	19.9981	0.001
		Lack of fit	9	3.10315×10^{-6}	344794	0.297825	0.919
		Pure error	2	2.31541×10^{-6}	1.15771×10^{-6}		

R^2, coefficient of correlation/goodness of fit; Q^2, goodness of prediction; *F*-value, Fischer's ratio; *p*-value, probability.

Supplementary Table S7. Regression equation coefficients for individual bioactive compounds in hazelnut involucre extracts.

Effect	Response													
	Y_1	Y_2	Y_3	Y_4	Y_5	Y_6	Y_7	Y_8	Y_9	Y_{10}	Y_{11}	Y_{12}	Y_{13}	Y_{14}
Constant	**2.3158**	**163.541**	**2.9321**	**60.6602**	**107.796**	**15.8665**	**4.1033**	**2.4923**	**36.7938**	**9.9322**	**84.9785**	**173.102**	**30.1594**	**4132.6**
X_1 (stirring time)	-0.0661	-4.8247	-0.3068	-2.5678	-4.1189	-1.2804	-0.2628	**-0.54002**	-0.0929	0.45407	-0.1204	2.6079	2.6029	310.19
X_2 (pH)	0.1786	4.4255	-0.1990	1.1239	-0.3423	0.8882	0.1804	0.3328	0.7309	-0.1229	1.3394	-6.7831	0.20101	-75.5127
X_3 (water in solvent)	**1.0532**	**61.553**	**0.70418**	**18.8426**	**40.4439**	**5.4662**	-0.4790	0.00229	**14.5517**	**4.6248**	**32.0497**	**-25.6402**	**-6.4099**	**-799.437**
X_3 x X_3	**-0.6276**	**-58.7996**	**-1.1265**	**-20.4025**	**-29.5338**	**-4.3143**	**-1.8433**	**-1.1561**	**-9.6238**	**-2.8291**	**-22.2799**	**-85.5579**	**-16.3786**	**-1570.93**
X_1 x X_2	-0.2968	-	-0.2878	-	-6.6490	-1.1849	-	-0.2718	-1.8656	-	-	-	-	-
X_2 x X_2	-	12.3175	-	-	-	-	-0.4836	-	-	-	-	-	-1.9819	-508.699
X_1 x X_3	-	-	-0.25007	-	-	-	-	-0.2806	-	0.9081	-	-4.0675	-	-
X_2 x X_3	-	-	-	-	-	-	-	0.2388	-	-	-	-	-	-
X_1 x X_1	-	-	-	-	-	-	-	-	-	0.9381	-	-14.0906	0.9479	-

Y_1 – Epicatechin; Y_2 – Catechin; Y_3 – Syringic acid; Y_4 – Gallic acid; Y_5 – Protocatechuic acid; Y_6 – Vanillic acid; Y_7 – *p*-Coumaric acid; Y_8 – Ferulic acid; Y_9 – Hyperoside; Y_{10} – Isoquercitrin; Y_{11} – Quercitrin; Y_{12} – Stigmasterol; Y_{13} – Campesterol; Y_{14} – Beta-sitosterol. For data in bold, p-value was <0.005, therefore statistically significant.

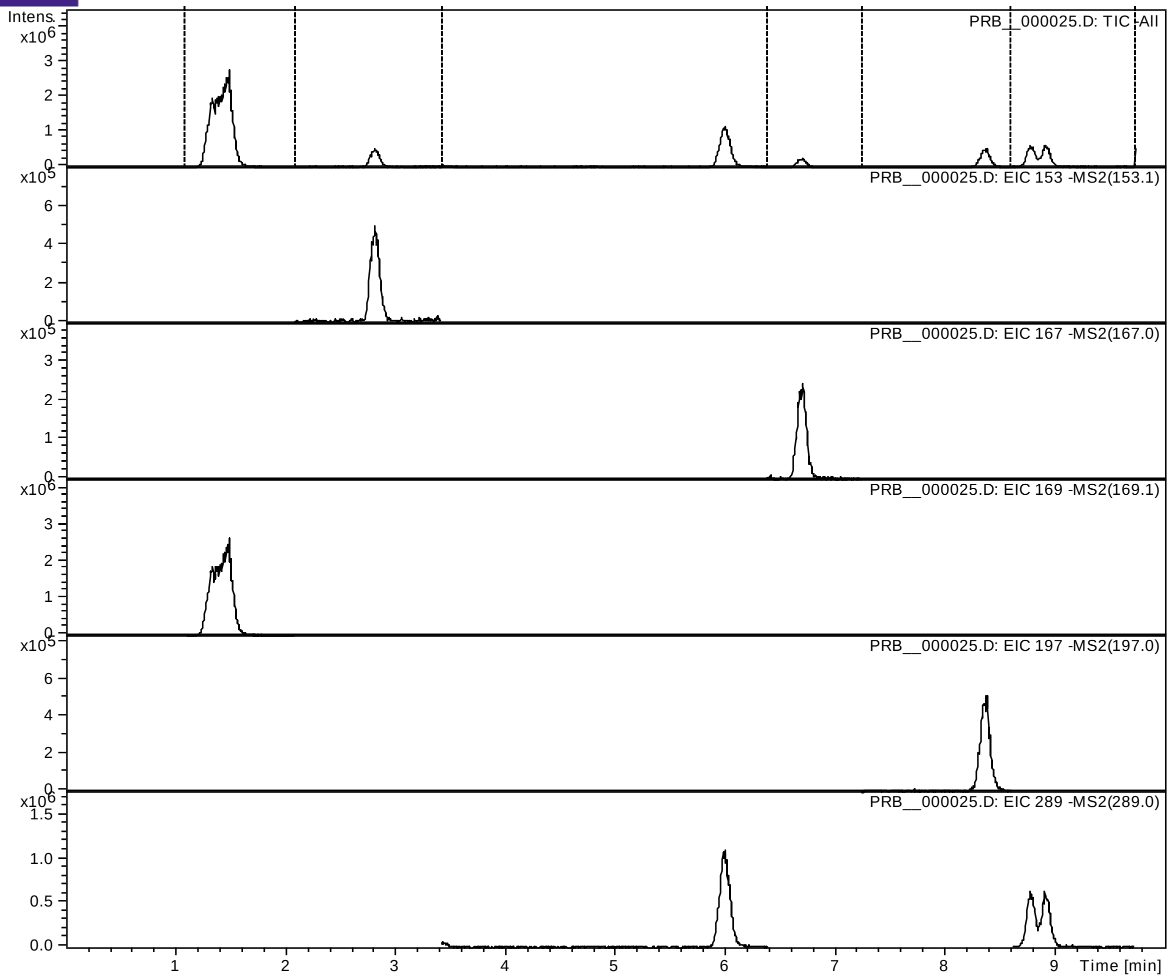

Supplementary Figure S1. The chromatograms for the individual polyphenolic compounds quantified by the LC-MS method II according to the retention time and m/z values in Supplementary Table 2.

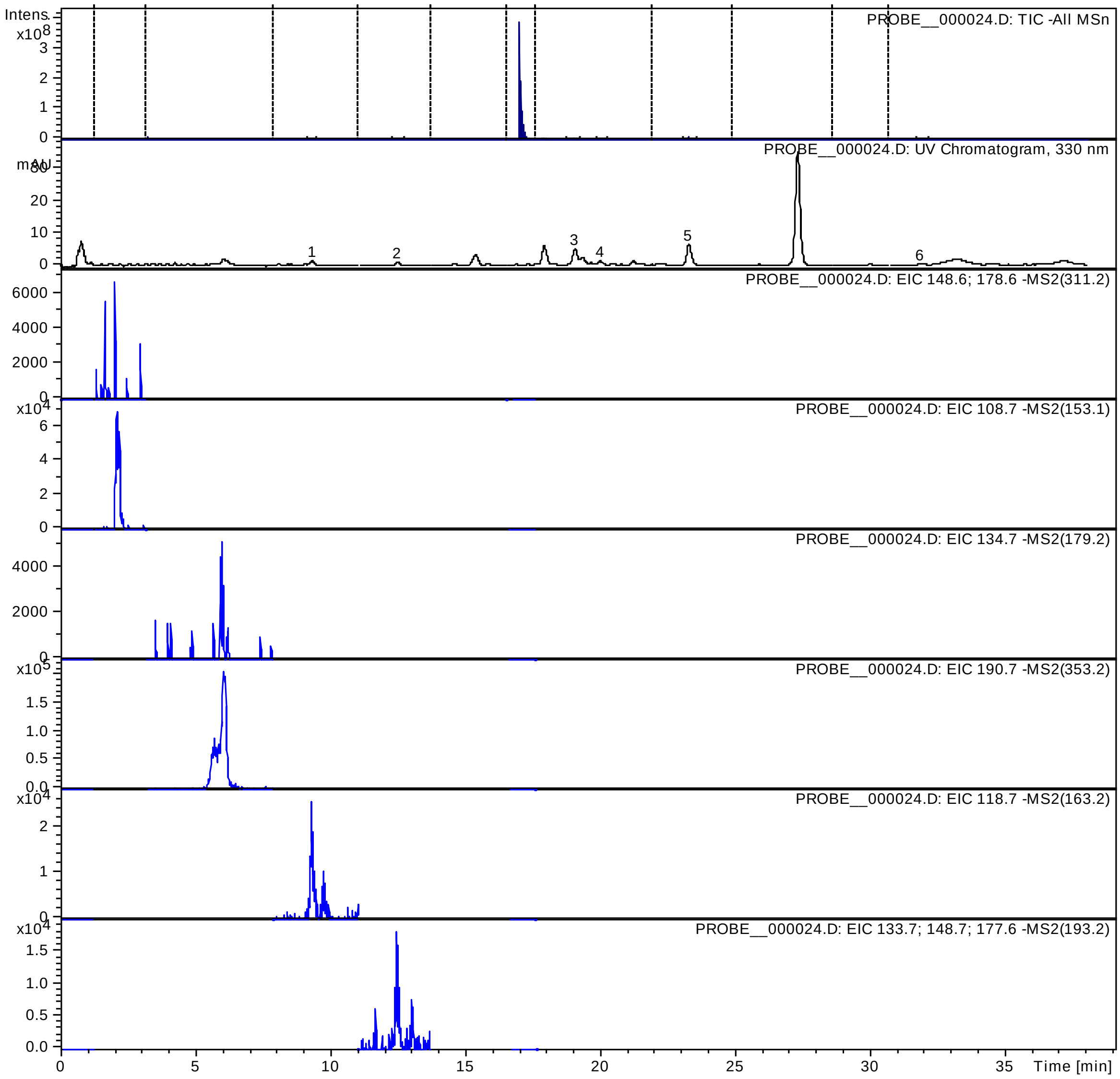

Supplementary Figure S2. The chromatograms for the individual polyphenolic compounds quantified by an LC-MS method according to the retention time and m/z values in Supplementary Table 1.

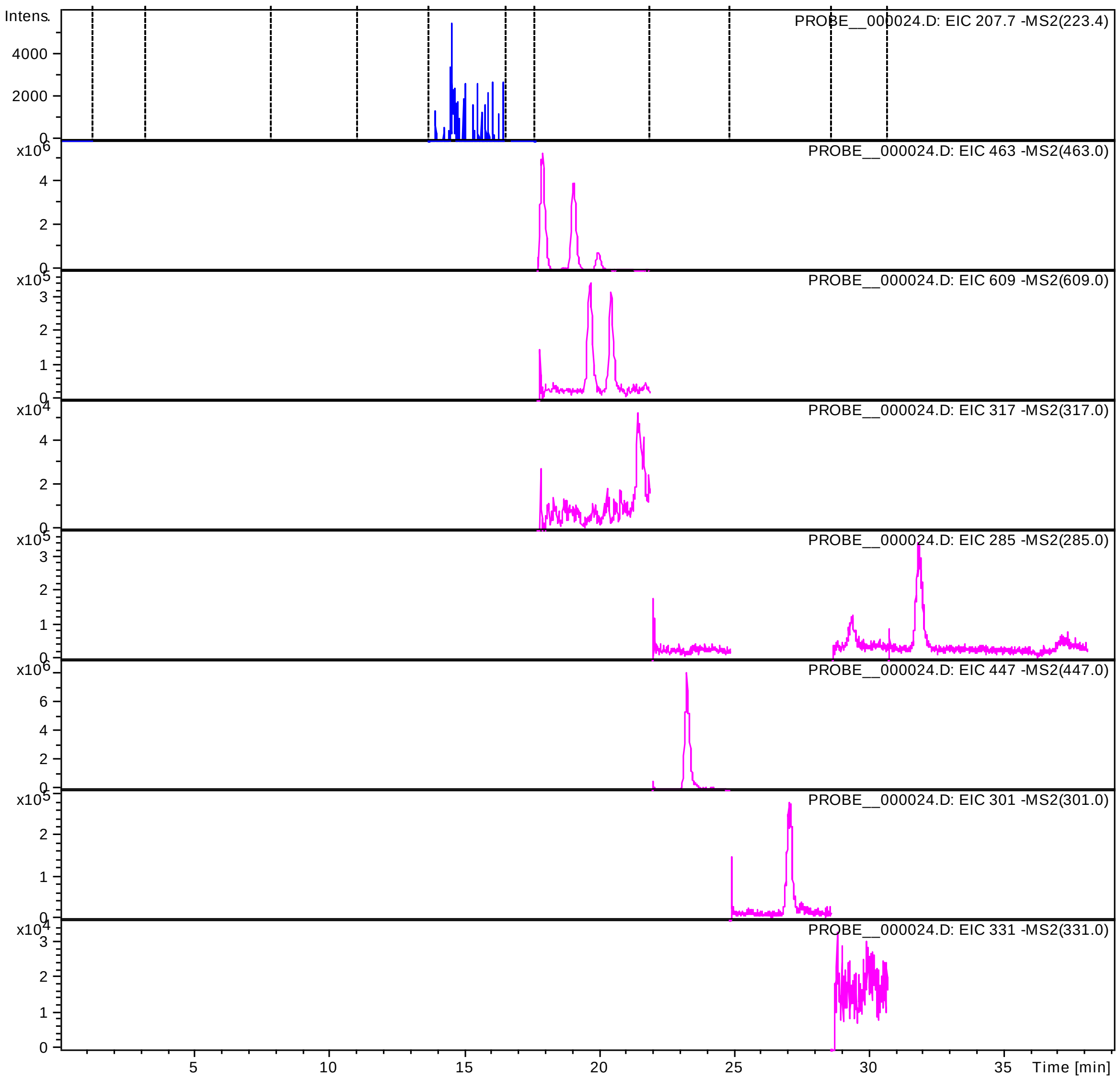

Supplementary Figure S3. The chromatograms for the individual polyphenolic compounds quantified by an LC-MS method according to the retention time and m/z values in Supplementary Table 1.

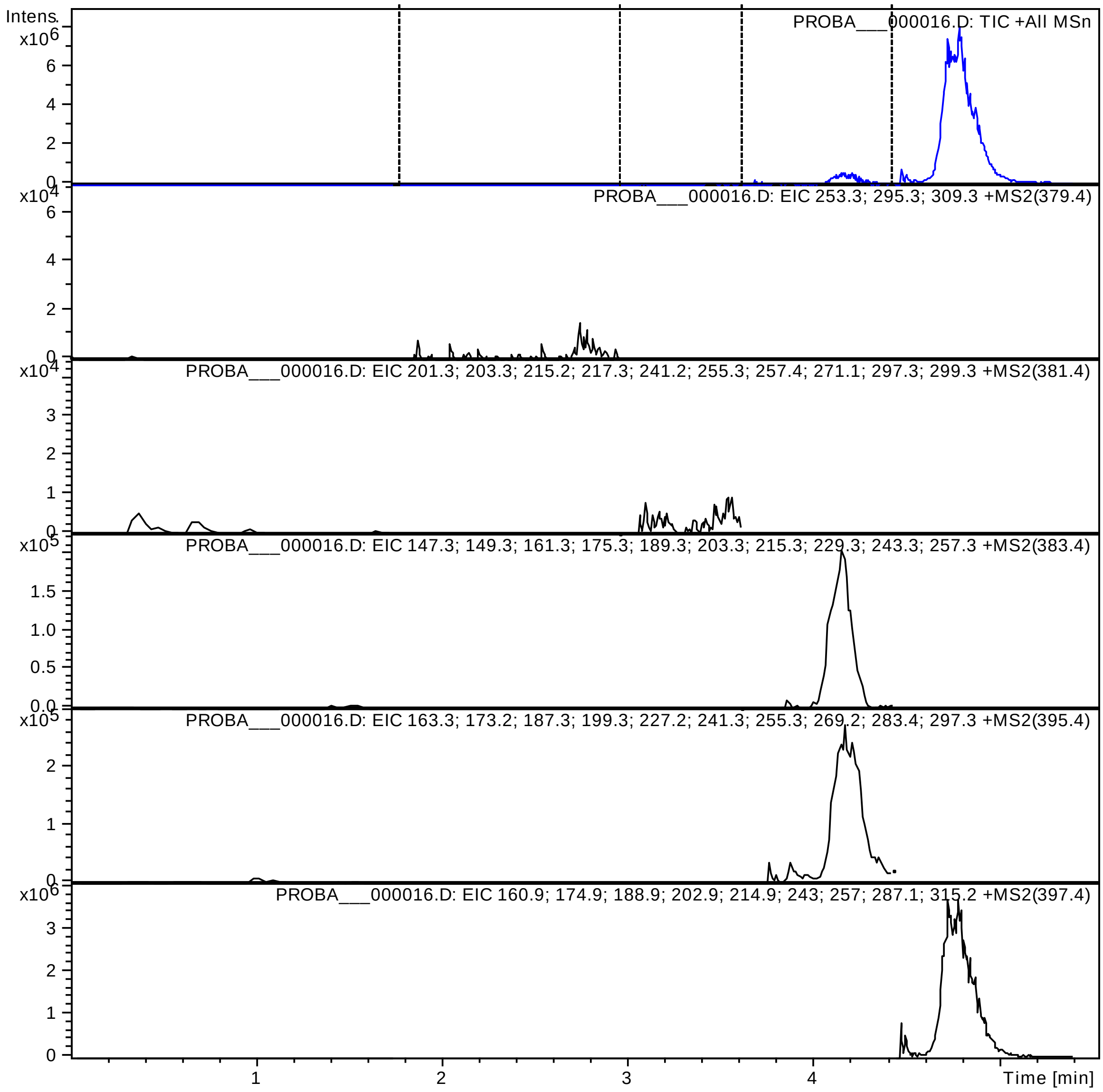

Supplementary Figure S4. The chromatograms for phytosterols quantified by an LC-MS/MS method according to the retention time and m/z values in Supplementary Table 3.
